# Mechanical Reinforced and Self‐healing Hydrogels: Bioprinted Biomimetic Methacrylated Collagen Peptide‐Xanthan Gum Constructs for Ligament Regeneration

**DOI:** 10.1002/adhm.202502341

**Published:** 2025-07-16

**Authors:** Hongjuan Weng, Monize Caiado Decarli, Lei He, Wen Chen, Sabine van Rijt, Katrien V. Bernaerts, Lorenzo Moroni

**Affiliations:** ^1^ Complex Tissue Regeneration Department MERLN Institute for Technology Inspired Regenerative Medicine Maastricht University Maastricht 6229 ER The Netherlands; ^2^ Sustainable Polymer Synthesis group Aachen‐Maastricht Institute for Biobased Materials Maastricht University Geleen 6167 RD The Netherlands; ^3^ Department of Biomaterials and Biomedical Technology University Medical Center Groningen University of Groningen Groningen 9713 AV The Netherlands; ^4^ Instructive Biomaterials Engineering Department MERLN Institute for Technology‐Inspired Regenerative Medicine Maastricht University Maastricht 6229 ER The Netherlands

**Keywords:** bioprinting constructs, collagen, methacrylated collagen peptide, self‐healing hydrogels, xanthan gum

## Abstract

Collagen peptide (COP) is water soluble, bioactive, and tends to be a promising alternative to collagen for tissue regeneration. However, its low viscosity and lack of readily polymerizable groups hinder its bioprinting and limit its wide applications in tissue engineering. In this study, methacrylated collagen peptide‐xanthan gum (COPMA‐XG) bioinks with interpenetrating networks are developed for bioprinting stable constructs, followed by stem cell differentiation. First, self‐healing COPMA hydrogels are developed with rapid UV‐curing and tunable mechanical properties. To increase the printability and the mechanical properties of COPMA, XG is mixed to create a set of COPMA‐XG bioinks. COPMA‐XG hydrogels show self‐healing properties, optimal printability, and stable morphology in the medium. The bioprinted human bone marrow mesenchymal stem cells (hMSCs) laden COPMA‐XG constructs are biocompatible and bioactive, with increased production of extracellular matrix, collagen type I, and scleraxis over 28 days. Overall, bioprinted COPMA‐XG constructs are versatile matrices to support hMSCs proliferation and differentiation with potential for ligament tissue engineering.

## Graphical abstract

Readily UV‐curing and self‐healing methacrylated collagen peptide (COPMA) hydrogels are developed with tunable mechanical properties. As the interpenetrating network between COPMA and xanthan gum (XG), COPMA‐XG bioinks show improved mechanical properties, printability, and stability, compared to COPMA or XG. The bioprinted stem cells laden COPMA‐XG constructs are biocompatible and biomimetic over 28 days, showing great potential for accelerating ligament regeneration.

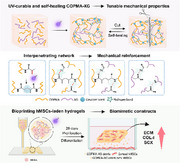

## 1 Introduction

Tissue engineering has attracted attention as an innovative approach to promote tissue reconstruction, particularly when using hydrogels for manufacturing bioprinted cell‐laden constructs. Hydrogels are highly hydrated 3D matrixes that support cell spreading, migration, and colonization.^[^ ^1^ ^]^ When cells are encapsulated in hydrogels to obtain cell constructs, ideal conditions for extracellular matrix (ECM) production, cell proliferation, and tissue formation can be achieved.^[^ ^2^, ^3^ ^]^ To manufacture cell constructs, bioprinting holds great potential since it enables the production of complex tissue architectures, with high control and reproducibility, resembling the natural design of the desired tissue.^[^ ^3^ ^]^

Collagen plays an important role in supporting the structure of ligaments with tensile strength and flexibility.^[^ ^4^ ^]^ Thus, collagen‐based hydrogels attracted considerable attention in ligament tissue engineering.^[^ ^5^ ^]^ However, collagen hydrogels often show low mechanical properties and low stability in aqueous solutions.^[^ ^6^, ^7^ ^]^ Their poor water solubility poses a problem for encapsulating cells in collagen solution, which requires advanced acidic solubilization and pH neutralization.^[^ ^8^ ^]^ Besides, their thermosensitive properties pose problems for precision bioprinting, which requires the use of cooling systems or supporting baths to avoid the unexpected gelation during printing.^[^ ^8^, ^9^, ^10^ ^]^

Collagen peptide (COP) is a mixture of low‐molecular‐weight (<6 kDa) peptides that are hydrolyzed from collagen by enzymatic digestion in acidic or alkaline condition.^[^ ^11^ ^]^ COP can be used as a water soluble and accessible collagen substitute material due to its outstanding bioactivity. It can activate endogenous collagen synthesis, with high moisture retention, antioxidant, and antimicrobial capacity.^[^ ^12^, ^13^ ^]^ Osteogenic and endothelial differentiation of rat bone marrow mesenchymal stem cells (rBMSCs) cultured in 2D in a COP supplement has also been shown.^[^ ^14^ ^]^ However, adipogenic and chondrogenic differentiation were inhibited in the same system. While culturing in a 3D culture system in a COP solution, adipose‐derived stromal cells (ADSCs) could differentiate for chondrogenic tissue.^[^ ^15^ ^]^ Nevertheless, the translation of COP to the clinics is currently limited to powder or solution administration forms, due to the inability of COP to form a hydrogel through self‐gelation.^[^ ^16^ ^]^ Hence, chemical modification of COP is essential to enable its gelation for broader applications in biomedicine.

The low viscosity of COP is another challenge that often impairs its processability when using standard bioprinting techniques.^[^ ^17^ ^]^ As reported by Geevarghese et al., COP was mixed with gelatin, alginate, and diethlamineorthyl cellulose. The blend was solidified by cooling at 4 °C before bioprinting, followed by post‐crosslinking in a calcium chloride solution.^[^ ^18^ ^]^ However, this cooling and post‐crosslinking procedure is time consuming, potentially cytotoxic to susceptible stem cells, and easily clotting the nozzle due to the heterogeneous gelation.^[^ ^19^ ^]^ Thus, there is a need for a more convenient method to smoothly bioprint COP based hydrogels.

**Figure 1:**
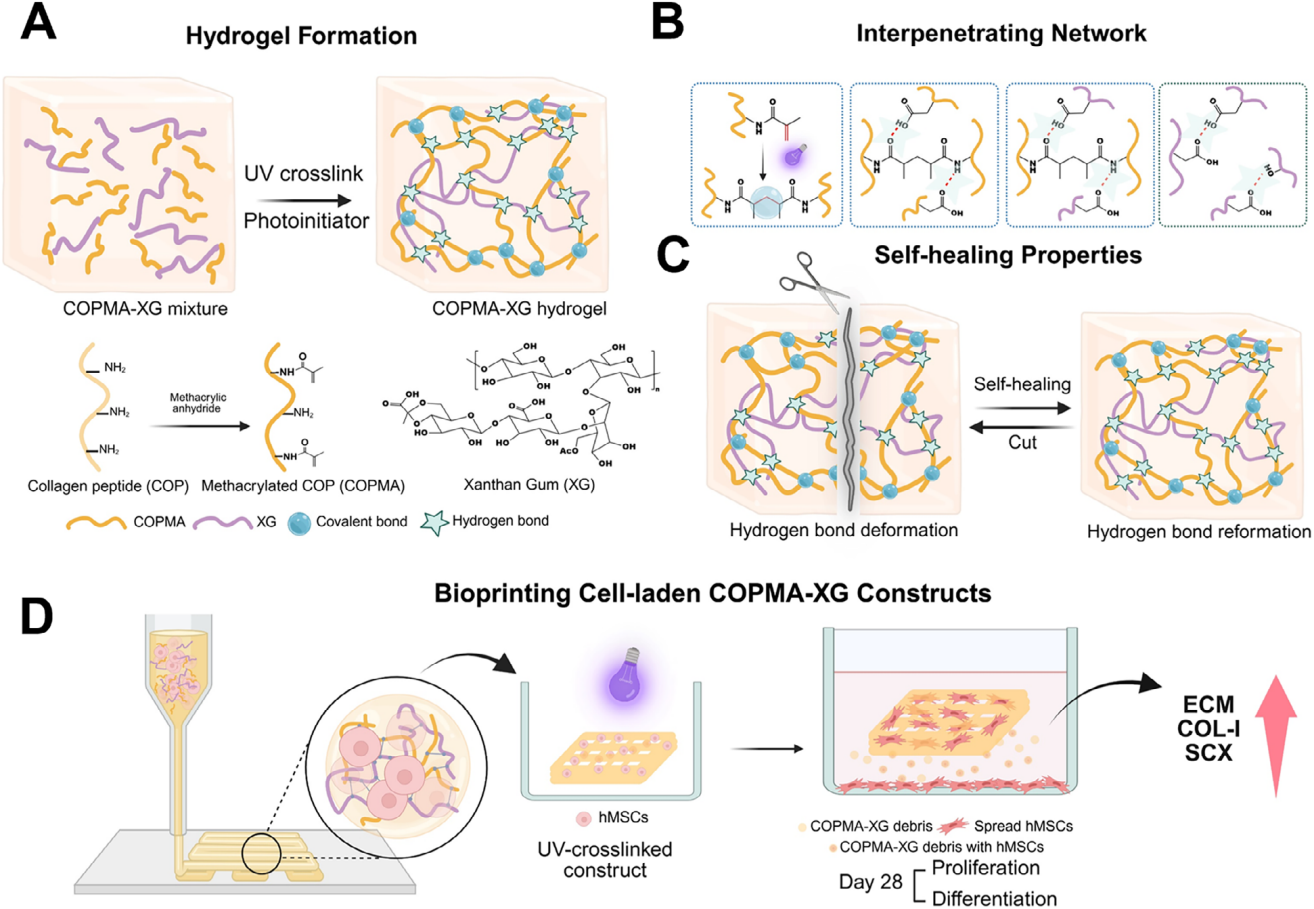
A) Synthesis of COPMA‐XG hydrogels. B) Interpenetrating network (covalent bonds and hydrogen bonds) in COPMA‐XG hydrogels. COPMA were covalently crosslinked under UV exposure. Hydrogen bonds existed between COPMA and XG. C) Self‐healing properties of COPMA‐XG hydrogels. D) hMSCs laden COPMA‐XG constructs were bioprinted and cultured in proliferation and differentiation media over 28 days. This figure was created with BioRender.com and has been granted a publication license.

In this study, we aim to develop a methacrylated collagen peptide‐xanthan gum (COPMA‐XG) bioink and establish a straightforward strategy for bioprinting it into stable constructs. First, COP was endowed with rapid photocrosslinking properties by synthesizing methacrylated collagen peptide (COPMA) (**Figure** **1**A). To improve printability of COPMA, we developed a novel COPMA‐based hydrogel by blending it with xanthan gum (XG) as a thickener.^[^ ^20^, ^21^ ^]^ XG is a natural polysaccharide, with good shape fidelity and biocompatibility.^[^ ^22^ ^]^ Additionally, intermolecular forces among the XG chains contribute to physical crosslinking,^[^ ^23^ ^]^ resulting in the formation of an interpenetrating network in COPMA‐XG consisting of both covalent bonds in UV‐crosslinked COPMA and hydrogen bonds between COPMA and XG (Figure 1B). The self‐healing property of COPMA‐XG hydrogels was studied (Figure 1C). The 3D printing parameters of COPMA‐XG were optimized, and the stability of the 3D printed hydrogel was investigated. Subsequently, hMSCs were encapsulated in the COPMA‐XG hydrogel for investigating proliferation and differentiation of hMSCs in the construct. Bioprinted constructs were obtained after UV curing and cell behavior was explored in constructs in proliferation and differentiation media over 28 days (Figure 1D).

## 2 Results

### 2.1 Synthesis of COPMA

**Figure 2:**
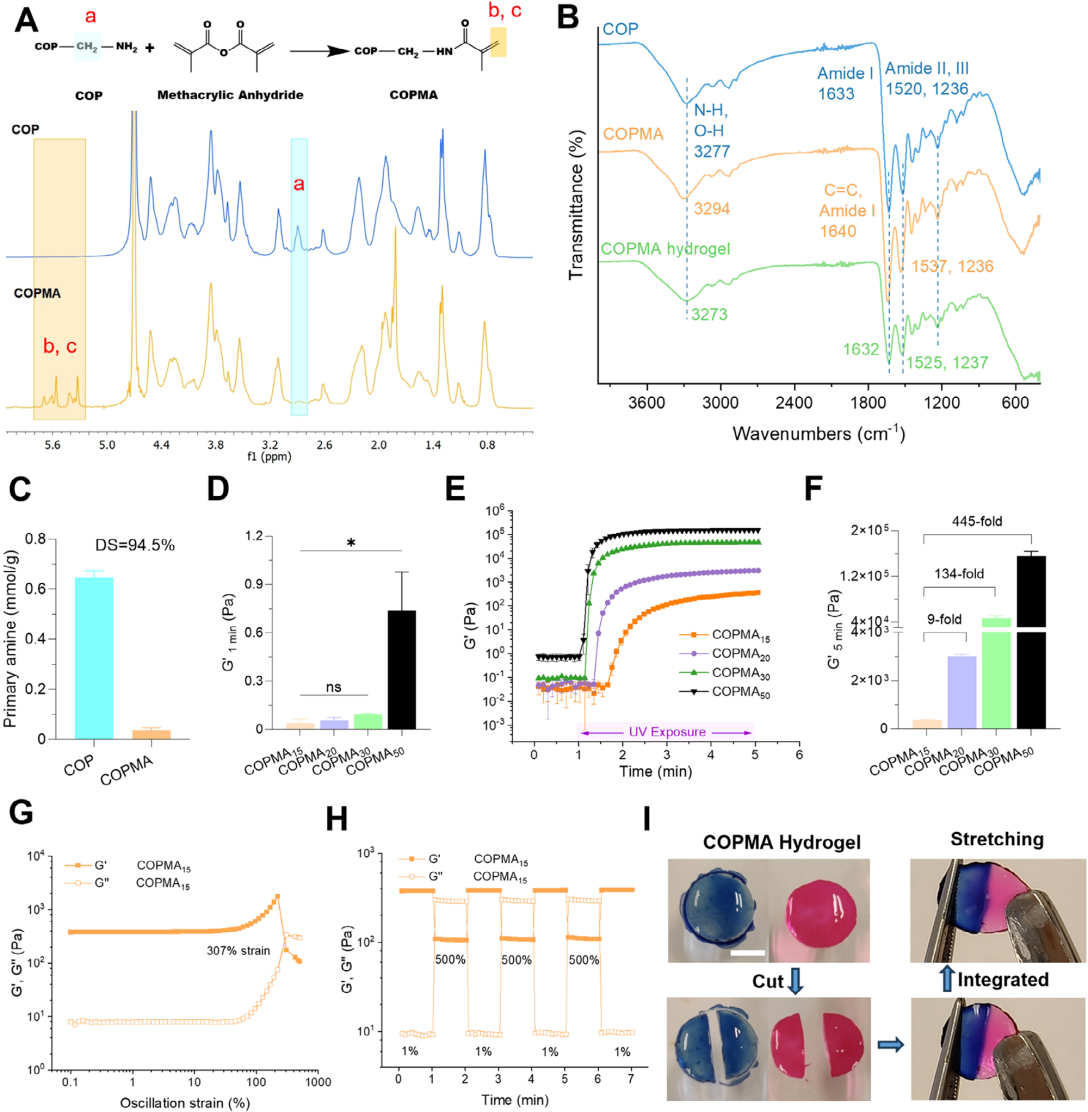
Characterizations of COPMA hydrogels. A) Reaction scheme and ^1^H NMR spectra of COP and COPMA. B) FTIR of COP, uncrosslinked COPMA, and UV‐cured COPMA hydrogels. C) Primary amine content in COP and COPMA as determined via the OPA assay. D) Storage modulus (G′) of COPMA hydrogel at 1 min (before UV irradiation), *n* = 2–3. E) Photorheology of COPMA hydrogel in various concentrations (15%, 20%, 30%, and 50% (w/v)). F) Storage modulus (G′) at 5 min (after UV irradiation). G) Strain‐dependent rheology of COPMA_15_ hydrogel during strain sweep from 0.1% to 500% at an angular frequency of 10 rad·s^−1^. H) Cyclic‐strain tests at low (1%) and high (500%) strains with a frequency of 10 rad·s^−1^. I) Self‐healing process of COPMA_15_ hydrogels, scale bar: 4 mm.

First, the water soluble and UV curable COPMA was synthesized by reacting COP with methacrylic anhydride. Modification of COP was examined by ^1^H NMR, FTIR, and spectrophotometric techniques. ^1^H NMR spectra confirmed the conversion of the primary amine groups in COP to methacrylamides (5.6 and 5.3 ppm) in COPMA (**Figure** **2**A). Besides, a considerable reduction of the proton next to the primary amine (─CH_2_NH_2_) at 2.89 ppm was observed in the COPMA compared to COP. In the FTIR image of COP, the amide I, II, and III peaks were shown at 1633, 1520, and 1236 cm^−1^, respectively (Figure 2B). In COPMA spectrum, the C═C stretching (methacrylate groups) peak overlapped with amide I at 1640 cm^−1^ and amide II peak was shifted to 1537 cm^−1^. The N–H and O–H stretching peaks were shifted from 3277 cm^−1^ (COP) to 3294 cm^−1^ (COPMA). From the spectrophotometric ortho‐phthalic dialdehyde (OPA) assay, the free primary amine in COP was 0.65 ± 0.02 mmol g^−1^ and 0.036 ± 0.01 mmol g^−1^ in COPMA. Thus, the degree of substitution (DS) of COPMA was 94.5%, indicating high modification efficiency (Figure 2C). The weight average molecular weight (M_w_) of COPMA was 3003 g mol^−1^, measured by aqueous gel permeation chromatography (GPC) (Figure S1A, Supporting Information).

Most collagen cannot dissolve in water and is unstable at room temperature because it can self‐assemble into triple‐helical fibers through numerous noncovalent interactions, such as electrostatic repulsion and hydrogen bonds.^[^ ^24^ ^]^ In contrast, COPMA, with its shorter polymer chains compared to collagen, exhibited limited self‐assembly, forming random linear clusters of nanoparticles without progressing to collagen‐like fibrils (Figure S1B, Supporting Information). This partial self‐assembly likely accounts for the water solubility and stability of COPMA at room temperature.

### 2.2 UV‐Curing COPMA Hydrogels

To determine the photocrosslinking kinetics and evaluate the mechanical properties of COPMA at various concentrations, an in situ photocrosslinking study was conducted in the linear viscoelastic region on the rheometer (Figure S2, Supporting Information). A series of COPMA hydrogels at concentrations of 15%, 20%, 30%, and 50% (w/v) were labeled as COPMA_15_, COPMA_20_, COPMA_30_, and COPMA_50_. COPMA hydrogels are formed by radical polymerization of methacrylamide under UV exposure. Before UV exposure, the storage modulus (G′) exhibited low values in COPMA hydrogels. (Figure 2D). Once UV irradiation started at 1 min, G′ and G′′ increased rapidly within 2 min, followed by a gradual stabilization, indicating high photocrosslinking efficiency (Figure 2E; Figure S3, Supporting Information). Notably, the mechanical properties of COPMA hydrogels were tunable by adjusting concentrations. Specifically, the storage modulus of COPMA hydrogel increased 9‐fold from 350 ± 35 Pa at 15% (w/v) to 3013 ± 81 Pa at 20% (w/v) and 134‐fold to 47 ± 3.7 kPa at 30% (w/v) after UV irradiation (Figure 2F). The storage modulus further increased to 156 ± 8.1 kPa with the increasing concentration to 50% (w/v). However, the COPMA_50_ showed a higher G′ than G′′ before UV exposure, indicating the unexpected self‐crosslinking (Figure 2E; Figure S3, Supporting Information). Besides, from the strain sweep, G′ and G′′ crossed at ≈307% strain, indicating the network deformation of COPMA_15_ hydrogel (Figure 2G). Dynamic amplitude tests (1% and 500% strain) were conducted, and photographs of cut/recombined hydrogels were conducted to verify self‐healing properties of the COPMA_15_ hydrogels (Figure 2H,I). When 500% strain was applied, the hydrogel network broke down (G′ < G′′). Once the strain was released to 1%, the G′ of COPMA_15_ hydrogel recovered to the initial value immediately, indicating its remarkable self‐healing capabilities (Figure 2H). Besides, two pieces of cut hydrogel could gradually merge to reform an integral hydrogel without external effect (Figure 2I). The self‐healing capacity of the COPMA hydrogel was attributed to hydrogen bonds, such as those formed between carboxyl and amide groups (Figure 1B). The formation of these hydrogen bonds was confirmed by the redshift of FTIR peaks. In FTIR of COPMA hydrogel, the peak corresponding to N–H and O–H stretching shifted from 3294 to 3273 cm^−1^, and amide I, II peaks shifted to 1632 and 1525 cm^−1^, when compared to the uncrosslinked COPMA (Figure 2B). In summary, COPMA hydrogels showed tunable stiffness with fast UV crosslink response within 2 min and showed self‐healing properties.

### 2.3 Printability of COPMA‐XG Hydrogels

**Figure 3:**
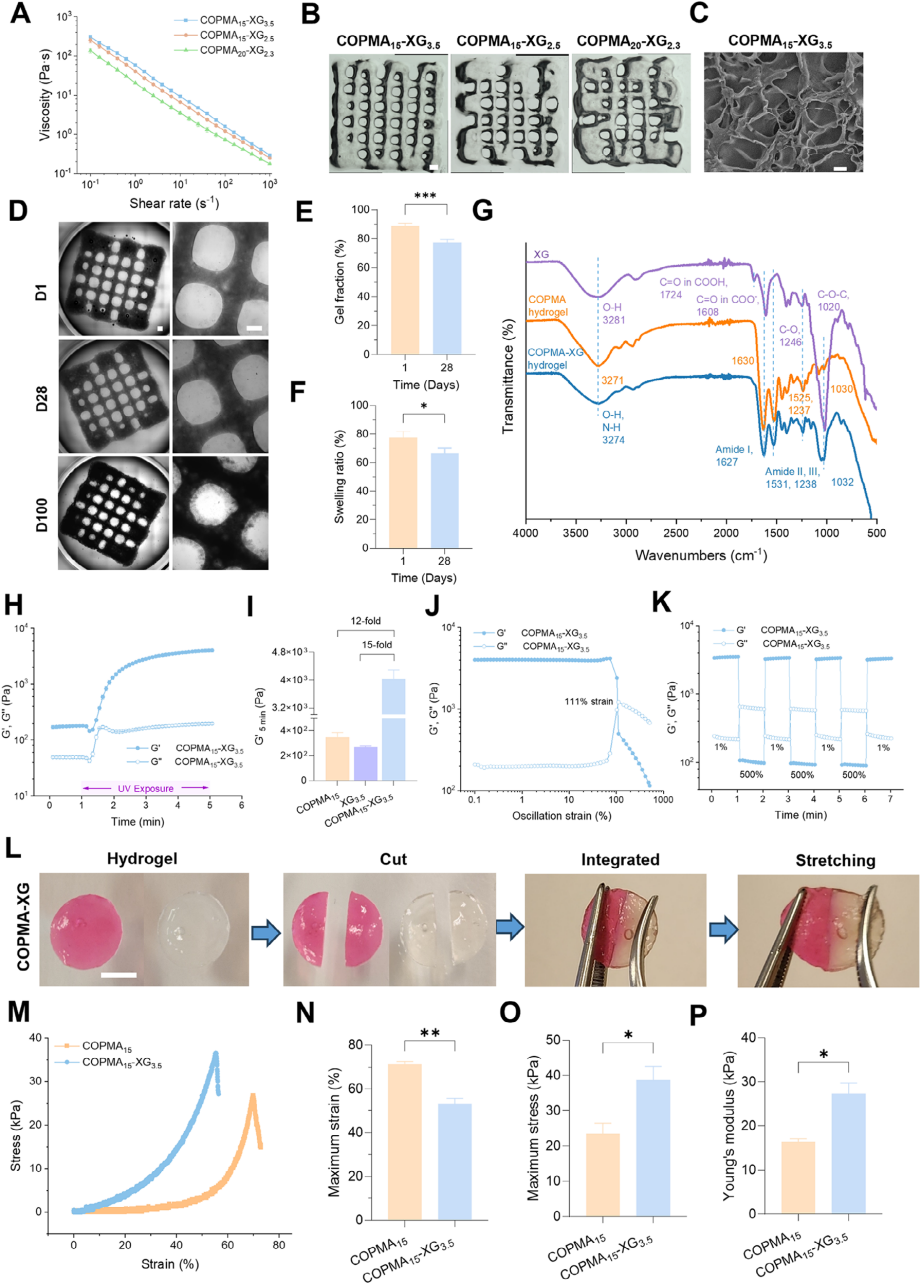
Characterization of COPMA‐XG hydrogels. A) Viscosity of COPMA‐XG in various mass ratios (COPMA_15_‐XG_3.5_, COPMA_15_‐XG_2.5_, and COPMA_20_‐XG_2.3_). B) Printability of COPMA‐XG with various ratios, scale bar: 1000 µm. C) SEM of COPMA_15_‐XG_3.5_, scale bar: 10 µm. D) Optical images of 3D printed COPMA_15_‐XG_3.5_ in medium for 100 days, scale bar: 500 µm. E,F) Gel fraction and swelling ratio of COPMA_15_‐XG_3.5_ hydrogels on day 1 and day 28 (n ≥ 3, *p < 0.05 and ***p < 0.001). G) FTIR of XG, COPMA hydrogel, and COPMA_15_‐XG_3.5_ hydrogel. H) Photorheology of COPMA_15_‐XG_3.5_ hydrogel. I) Comparison of storage modulus (G′) at 5 min. J) Strain‐dependent rheology of COPMA_15_‐XG_3.5_ hydrogel during strain sweep from 0.1% to 500% at an angular frequency of 10 rad·s^−1^. K) Cyclic‐strain tests of COPMA_15_‐XG_3.5_ hydrogel at low (1%) and high (500%) strains with frequency of 10 rad·s^−1^. L) Self‐healing process of COPMA_15_‐XG_3.5_ hydrogel, scale bar: 4 mm. M) Representative compressive stress–strain curve of COPMA_15_ and COPMA_15_‐XG_3.5_ hydrogels. N,O) Maximum strain and stress from compressive tests. P) Young's modulus of COPMA_15_ and COPMA_15_‐XG_3.5_ hydrogels. (n ≥ 3, *p < 0.05 and **p < 0.01).

Although COPMA contains UV‐curable groups, its low viscosity still poses challenges for bioprinting applications (Figure S4A,D, Supporting Information). Thus, biocompatible XG was blended with COPMA as a thickener to improve printability. The COPMA‐XG bioink could be injected smoothly from a 30 G nozzle (0.159 mm diameter) and a 25 G nozzle tip (0.25 mm diameter) (Figure S4B,C, Supporting Information). A series of COPMA‐XG mixtures varying in mass ratios of COPMA and XG were investigated to optimize bioink formulations (Figure S5, Supporting Information). Specifically, three formulations (COPMA_15_‐XG_3.5_, COPMA_15_‐XG_2.5_ and COPMA_20_‐XG_2.3_, with the subscripts representing wt%) passed the vial inversion test (Figure S5C–E, Supporting Information), which is a simple well‐established test to demonstrate the most promising materials for bioprinting.^[^ ^20^ ^]^ Thus, these three formulations were further 3D printed to investigate the optimal printing parameters. The printing parameters of COPMA_15_‐XG_3.5_ hydrogel were first optimized, resulting in favorable printing speed of 35–40 mm s^−1^ and pressure of 40–45 kPa (Figure S6 and Table S1, Supporting Information). However, because of more COPMA mass ratio in COPMA_15_‐XG_2.5_ and COPMA_20_‐XG_2.3_, these viscosities decreased significantly when compared to COPMA_15_‐XG_3.5_ (**Figure** **3**A; Figure S7A, Supporting Information). Consequently, higher speed and lower pressure were necessary to get homogenous filaments (Table S1, Supporting Information). Even with the optimized printing parameters, the morphologies of COPMA_15_‐XG_2.5_ and COPMA_20_‐XG_2.3_ hydrogels were less homogenous than COPMA_15_‐XG_3.5_ hydrogel (Figure 3B). Therefore, the COPMA_15_‐XG_3.5_ hydrogel was selected for further studies. The 3D printed COPMA_15_‐XG_3.5_ hydrogel showed a porous structure (Figure 3C; Figure S7B, Supporting Information) similar to other hydrogels,^[^ ^25^ ^]^ which may further benefit the cell interaction in the hydrogel via facilitating cell signaling and nutrient exchange.

### 2.4 Stability of COPMA_15_‐XG_3.5_ Hydrogels

The stability of scaffolds was assessed by optical microscopy, gel fraction, and swelling ratio. The optical images revealed that COPMA_15_‐XG_3.5_ hydrogel slowly degraded and released some debris in the medium but kept an organized morphology within 100 days at 37 °C (Figure 2D). However, the XG_3.5_ gradually dissolved in the medium in 1 day (Figure S8, Supporting Information). The gel fraction and swelling ratio statistically revealed the stability of the hydrogels. Because the XG_3.5_ gradually dissolved in the aqueous solution, the gel fraction and swelling ratio of XG_3.5_ could not be detected. However, the COPMA_15_‐XG_3.5_ hydrogels showed high gel fraction (88.9% ± 1.5%) on day 1, indicating a well crosslinked network (Figure 3E). The swelling ratio of COPMA_15_‐XG_3.5_ hydrogels in PBS was 77.4% ± 4.0% at day 1, demonstrating its high hydrophilicity, good moisture absorption, and retention capacity (Figure 3F). These high gel fraction and swelling ratio of the COPMA_15_‐XG_3.5_ hydrogel may be attributed to the interpenetrating network consisting of covalent bonds and hydrogen bonds (Figure 1B; Figure 3G). Because of the strong hydrogen bonds, a broad peak shifted from 3281 cm^−1^ (XG, O–H stretching) to 3274 cm^−1^ (COPMA‐XG, O–H and N–H stretching). Besides, the amide I peak shifted from 1630 cm^−1^ (COPMA hydrogel) to 1627 cm^−1^ (COPMA‐XG hydrogel) (Figure 3G). Due to the degradation over 28 days, the swelling ratio decreased to 66.5% ± 3.2%. Similarly, its gel fraction significantly dropped to 77.2% ± 2.2%, resulting in 22.8% degradation over 28 days. Overall, the gradual degradation properties of COPMA_15_‐XG_3.5_ hydrogel might facilitate cell delivery and aggregation for accelerating tissue repair.^[^ ^26^ ^]^

### 2.5 Mechanical Properties of COPMA_15_‐XG_3.5_ Hydrogels

The mechanical properties of COPMA_15_‐XG_3.5_ hydrogels were investigated by rheological and compressive tests. First, the UV curing efficiency and mechanical properties of XG_3.5_ and COPMA_15_‐XG_3.5_ hydrogels were studied by in situ photorheology in the linear viscoelastic region (Figure S9, Supporting Information). Due to the lack of UV‐curable functional groups, no significant increase in the storage modulus (G′) of XG_3.5_ was observed during UV irradiation (Figure S10, Supporting Information). Conversely, once UV irradiation was initiated at 1 min, G′ of COPMA_15_‐XG_3.5_ hydrogels demonstrated a rapid increase within 3 min, followed by a gradual stabilization (Figure 3H). Specifically, the G′ of COPMA_15_‐XG_3.5_ hydrogels increased from 180 ± 9 Pa (G′_1 min_) to 4027 ± 264 Pa (G′_5 min_) after UV exposure (Figure 3H). This indicated that the COPMA_15_‐XG_3.5_ hydrogels could be UV cured within 3 min due to the methacrylate triggered radical polymerization (Figure 1A,B). Attributed to the synergistic effect of chemical and physical crosslinking via covalent bonding and hydrogen bonding, the G’_5 min_ of COPMA_15_‐XG_3.5_ hydrogels was 15‐fold higher than G’_5 min_ of XG_3.5_ (270 ± 8 Pa) and 12‐fold higher than G’_5 min_ of COPMA_15_ (350 ± 35 Pa) after the UV irradiation (Figure 3I).

Dynamic mechanical properties of COPMA_15_‐XG_3.5_ hydrogels were characterized by strain sweep and cyclic strain tests. From the strain sweep (0.1–500%), COPMA_15_‐XG_3.5_ hydrogels maintained the viscoelastic state until strain up to 111% (Figure 3J). Thus, the cyclic strain tests were alternatively conducted at 1% and 500% strain. The hydrogel network was disrupted (G′ < G′′) under high strain (500%). Once the strain was released to 1%, it reverted to gel phase by recovering to its initial G′ value rapidly (Figure 3K). In the photographs of hydrogel deformation/reformation, when two pieces of hydrogel were brought in contact, they gradually and spontaneously reformed into an integral hydrogel. Dye diffusion (Rhodamine B in red) was visible at the interface between 2 cut pieces of hydrogels (Figure 3L). The integrated hydrogel could resist a certain strength without interfacial separation. These tests indicated self‐healing properties of COPMA_15_‐XG_3.5_ hydrogels, due to the deformation and reformation of the dynamic hydrogen bonds.

From the compressive stress‐strain curves of the hydrogels, the maximum strain of COPMA_15_ and COPMA_15_‐XG_3.5_ was 71.4 ± 0.97% and 53.3 ± 2.0%, respectively (Figure 3M,N). COPMA_15_‐XG_3.5_ hydrogels exhibited a higher maximum stress (38.7 ± 3.2 kPa) than COPMA_15_ hydrogels (23.5 ± 2.5 kPa) (Figure 3O). The Young's modulus increased from 16.4 ± 0.6 kPa (COPMA_15_) to 27.4 ± 2.0 kPa (COPMA_15_‐XG_3.5_) (Figure 3P). Overall, the introduction of XG increased the mechanical properties of COPMA_15_‐XG_3.5_ hydrogels due to their interpenetrating network, when compared to COPMA_15_ hydrogels (Figure 1B).

### 2.6 Bioprinting of hMSCs Laden COPMA_15_‐XG_3.5_ Hydrogels and Cell Viability

**Figure 4:**
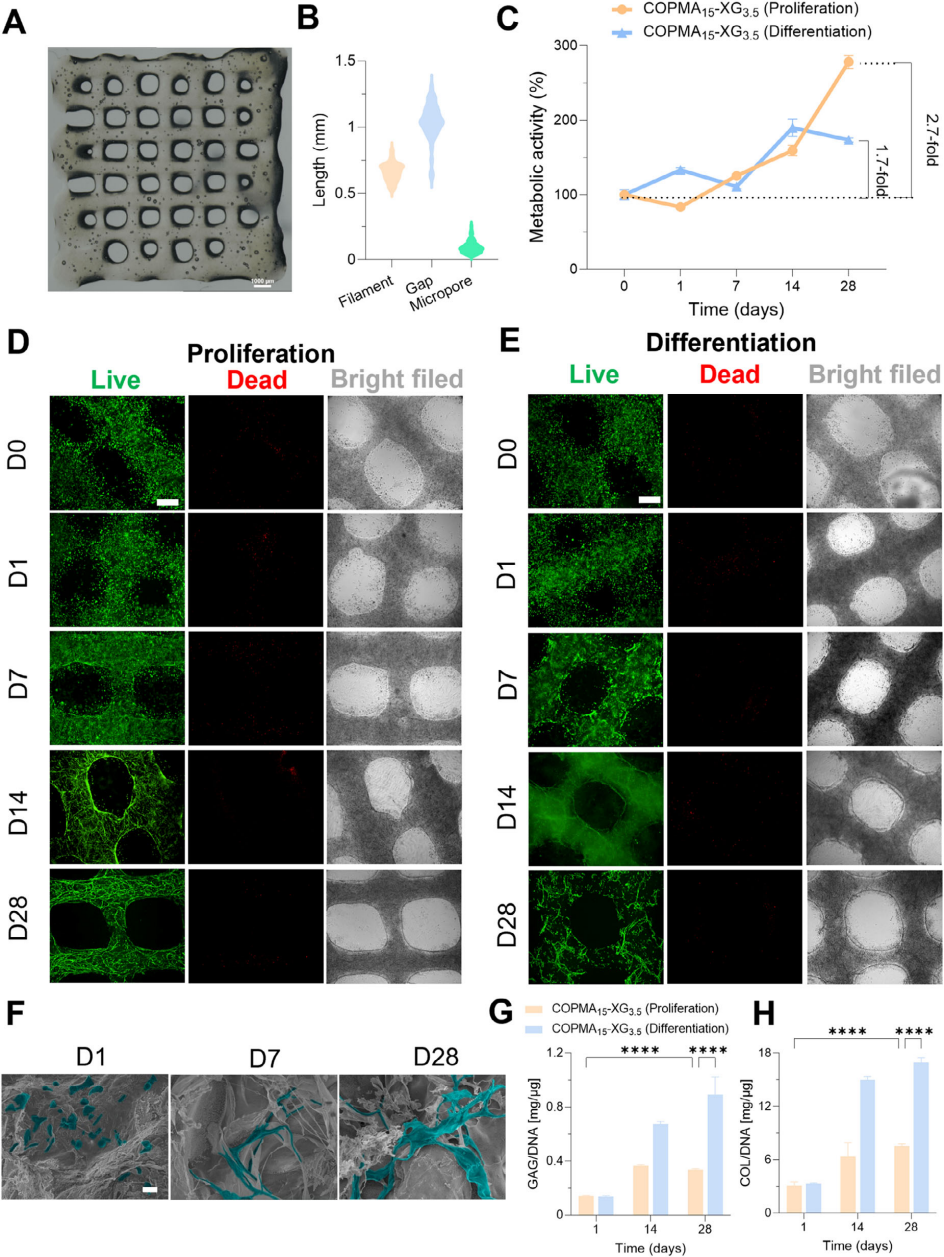
Biocompatibility of hMSCs laden COPMA_15_‐XG_3.5_ constructs. A) Optical image of bioprinted hMSCs laden COPMA_15_‐XG_3.5_ hydrogel. B) Size statistics of bioprinted constructs. C) Metabolic activity. D‐E) Live/dead staining of hydrogels in proliferation medium, and in differentiation medium, scale bar: 500 µm. F) SEM of hMSC encapsulated COPMA_15_‐XG_3.5_ hydrogel on day 1, day 7, and day 28 at ×500 magnification. The blue highlights indicate the presence of hMSCs. Scale bar: 20 µm. G,H) GAG/DNA and COL/DNA in hMSC encapsulated COPMA_15_‐XG_3.5_ hydrogels on day 1, 14, and 28 (n ≥ 3, ****p < 0.0001).

Bioprinting enables precise deposition of numerous cells in hydrogels with organized morphology in a single step.^[^ ^27^ ^]^ Thus, to investigate the potential of COPMA‐XG hydrogels in tissue regeneration, hMSCs laden hydrogels were bioprinted into 3‐layered constructs (10 mm × 10 mm, wide × length). These constructs showed homogenous filaments (0.66 ± 0.07 mm) with micropore diameters (95 ± 45 µm) and gaps (1.04 ± 0.13 mm), which may facilitate cell‐cell interactions and nutrient penetration (**Figure** **4**A,B).

The biocompatibility of bioprinted hMSCs constructs was evaluated by metabolic activity and live/dead staining in proliferation and ligament differentiation media. Overall, an increase in metabolic activity was detected in cell‐laden COPMA_15_‐XG_3.5_ hydrogels over time in both culture media, indicating good biocompatibility of bioprinted constructs. Compared to day 0, the metabolic activity increased 2.7 times and 1.7 times on day 28 in proliferation and differentiation media, respectively (Figure 4C). This may be because stem cells were committed to proliferation in the early period and differentiation toward ligament cells at a later stage.

hMSCs showed high viability after bioprinting, as evidenced by the presence of predominantly live cells (Figure 4D,E; Figure S11A, Supporting Information). Correspondingly, cells gradually spread and proliferated with high cell viability over 28 days in proliferation medium (Figure 4D). hMSCs showed less density and different cell phenotype in the differentiation medium at day 28 compared to the proliferation medium (Figure 4E), suggesting the ongoing differentiation. From SEM analysis, hMSCs spread and formed longer protrusions in the bioprinted cell encapsulated constructs over time (Figure 4F; Figure S11B, Supporting Information), indicating a good interaction between cells and hydrogels.

### 2.7 Quantification of ECM Production

To investigate the ECM production in bioprinted constructs, two main ECM components (glycosaminoglycan and total collagen) were analyzed. First, DNA content in the hydrogel was detected in a decreased trend from day 1 to day 28, because of cell migration from hydrogels to the well plate (Figure 4D,E; Figure S12, Supporting Information). This effect was more pronounced in the differentiation media on day 28, probably because cells were more inclined to produce ECM. After normalization to DNA content, glycosaminoglycan (GAG) increased from day 1 to day 28 in both groups (Figure 4G). GAG/DNA in constructs under differentiation conditions increased from day 1 to day 14, followed by a slow increase to day 28. A significant increase in collagen/DNA over time was observed in both conditions, indicating high efficiency in collagen production on day 28. The collagen/DNA ratio was higher in the differentiation condition than in the proliferation condition on day 28, indicating the better ECM modeling capacity (Figure 4H).

### 2.8 hMSCs Differentiation in Constructs

Collagen type I (COL‐I) is one of the most abundant proteins in ECM and main component in ligaments.^[^ ^5^ ^]^ Scleraxis (SCX) is a highly specific ligament marker, acting as a critical transcription factor for ligament development.^[^ ^28^ ^]^ Transforming growth factor‐beta 3 (TGF‐β3) is one of ligament/tendon inducing growth factors,^[^ ^29^ ^]^ which was supplemented in the differentiation medium but not in proliferation medium. The versatility of bioprinted constructs was evaluated by culturing in proliferation and differentiation media with the quantification of COL‐I and SCX antibodies.

**Figure 5:**
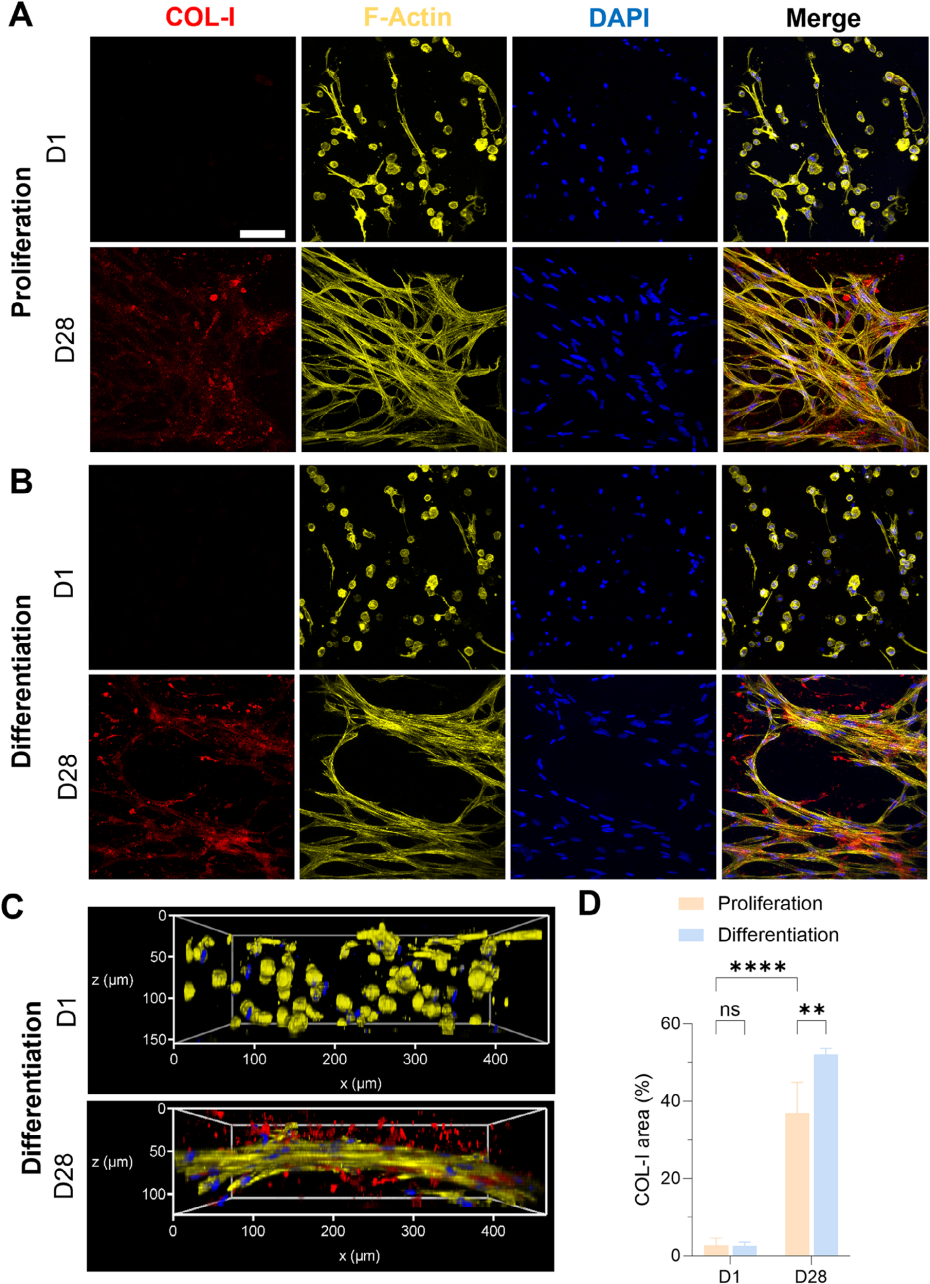
Immunostaining of COL‐I expression in hMSCs laden COPMA_15_‐XG_3.5_ hydrogels. A) In proliferation medium. B) In ligament differentiation medium. C) 3D reconstruction on day 1, and day 28 of the cellular network in hydrogels in differentiation condition. Scale bar: 100 µm. D) Relative fluorescence COL‐I area (%) in the constructs on day 1 and 28 (n ≥ 3, ns indicates not siginificant, * *p *> 0.05, **p < 0.01, ****p < 0.0001).

**Figure 6:**
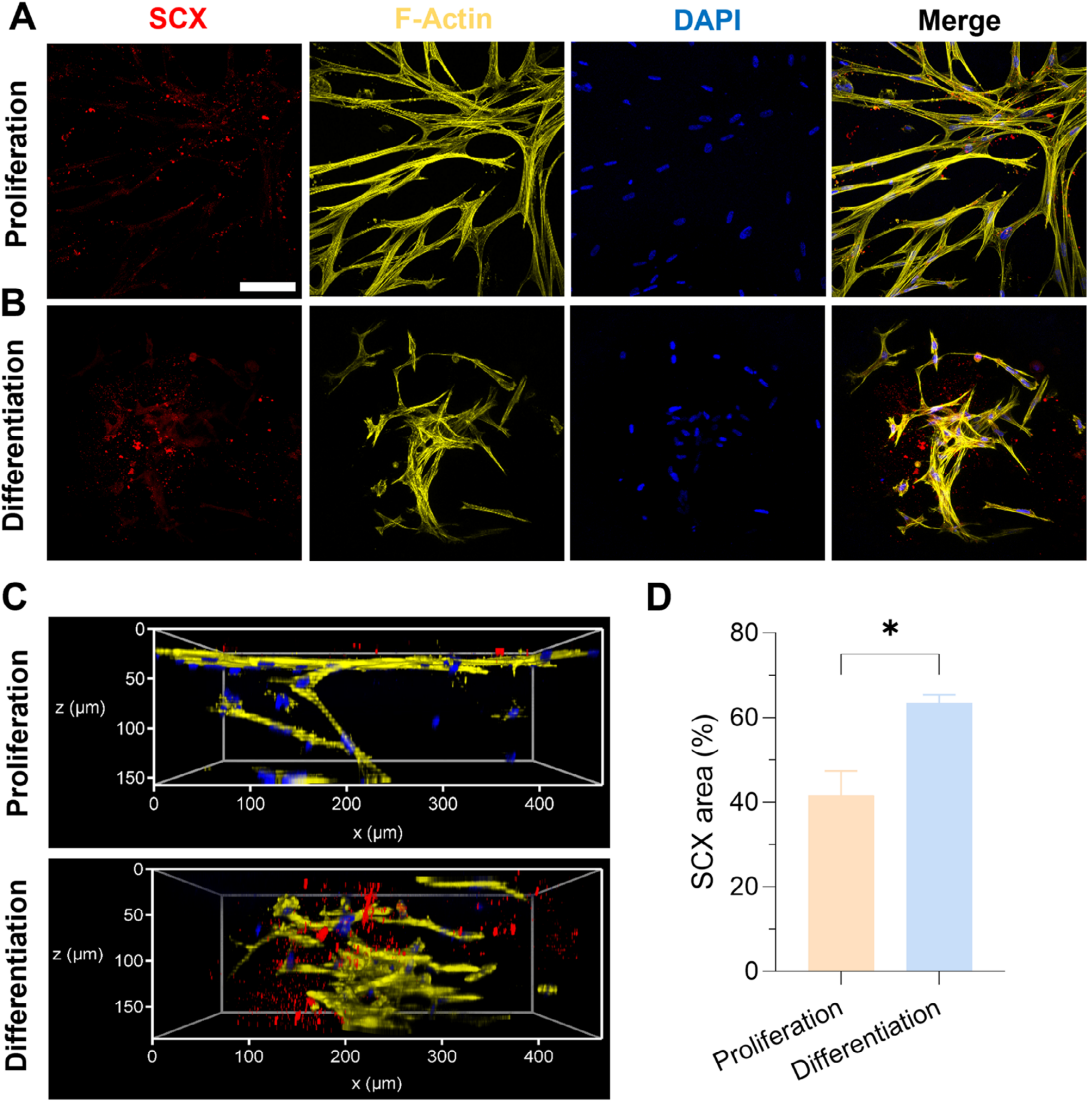
Immunostaining of SCX expression in hMSCs laden COPMA_15_‐XG_3.5_ hydrogels on day 28. A) In proliferation medium. B) In differentiation medium. Scale bar: 100 µm. C) 3D reconstruction of the cellular network in hydrogels in proliferation medium (top) and differentiation medium (bottom). D) Relative fluorescence SCX area (%) in the constructs on day 28 (n ≥ 3, *p < 0.05).

hMSCs were homogeneously embedded in the bioprinted constructs and started to elongate, yet without significant COL‐I expression at day 1 (**Figure** **5**A,B, top). On day 28, cells formed a 3D interconnected network in both media (Figure 5C) and displayed a higher expression of COL‐I in differentiation medium (Figure 5A,B, bottom, and Figure 5D). This indicated good intercellular communication in the hydrogel and a more abundant ECM deposition under differentiation conditions. The SCX marker was observed under proliferation conditions (**Figure** **6**A), which may be because constructs provided a stable 3D collagen peptides‐rich microenvironment to facilitate the sustained cell‐matrix interactions and deposit of ECM and bioactive markers. Collagen peptides have been reported to promote cells producing endogenous collagen by activating the TGF‐β/Smad signaling pathway.^[^ ^30^ ^]^ These constructs with abundant collagen peptides may act as biochemical cues to promote cells to produce ECM, COL‐I, and SCX, which further stimulate cell differentiation toward ligamentocytes. Notably, more SCX was expressed in the differentiation group, probably due to the synergetic effect between bioactive constructs and TGF‐β3 in the differentiation medium (Figure 6D). Simultaneous expressions of COL‐I and SCX on day 28 confirmed the differentiation of hMSCs into ligamentocytes in constructs.

### 2.9 Bioprinting of hMSCs Laden COPMA_20_‐XG_3.5_ Constructs

To investigate the effect of higher COPMA concentration on the mechanical properties and cytocompatibility, hMSCs‐laden COPMA_20_‐XG_3.5_ hydrogels were also bioprinted (Table S2, Supporting Information). Because of the same volume ratio of COPMA and XG (1:1), the same good shape fidelity as COPMA_15_‐XG_3.5_ constructs was observed (Figure S13, Supporting Information). After UV curing, the COPMA_20_‐XG_3.5_ hydrogel exhibited 2.4‐fold higher storage modulus (11.4 ± 2.4 kPa) than COPMA_15_‐XG_3.5_ hydrogel (Figure S14, Supporting Information), indicating stiffer and more condensed networks. The bioprinted constructs showed good biocompatibility with the majority of live stem cells, but only a few cells spread in the construct or migrated to the well plate over 28 days. (Figure S15A,B, Supporting Information). In addition, the metabolic activity and DNA content of hMSCs in the COPMA_20_‐XG_3.5_ constructs kept stable over 28 days (Figure S15C,D, Supporting Information). This indicated that the COPMA_20_‐XG_3.5_ constructs showed good cell retention, and a lower capacity of cell mediated remodeling over 28 days. This is likely due to the embedded hMSCs trapped into a tighter hydrogel network, which limited cell‐cell interactions and diffusion efficiency of nutrients and wastes, further hindering their spreading and migration. Thus, cell behavior in the bioprinted COPMA‐XG constructs could be modulated by easily adapting the concentration of COPMA resulting in tunable mechanical properties. Such stiffer COPMA‐XG constructs could find applications in articular cartilage tissue engineering, for example, where the retention of a more rounded cell morphology is pivotal to functional tissue regeneration.

## 3 Discussion

Bioprinted cell‐laden hydrogels are attracting increasing attention because they can act as an artificial ECM to support cell proliferation in vitro. Besides, the bioprinted constructs can mimic the complex structure and components of tissues, showing good potential in tissue regeneration.^[^ ^3^ ^]^ Collagen, as a main component in ECM, has been widely used as a platform for cell proliferation. However, the low thermostability and poor water solubility of collagen limit the bioprinting cell‐laden collagen hydrogels.^[^ ^31^ ^]^ COP was developed as collagen alternative, with good water solubility.^[^ ^32^ ^]^ The COP remains soluble and flowable in body fluid at physical temperature, enabling its bioprinting at room/physical temperature. In this study, we aim to bioprint hMSCs laden COPMA‐XG hydrogels with self‐healing, tunable mechanical properties, and good bioactivity.

First, a novel photocurable COPMA hydrogel with tunable mechanical properties was successfully synthesized. COP itself failed to form a hydrogel due to its highly hydrophilic nature and lack of readily polymerizable groups. One of the strategies to develop COP hydrogels has been reported by copolymerizing with other polymers.^[^ ^33^ ^]^ For instance, COP was coupled with sodium alginate (SA), then COP‐SA hydrogels were formed by bioprinting into a calcium chloride support bath.^[^ ^34^ ^]^ However, these COP hydrogels are not stable for a long‐term due to the coordination bonds between COP‐SA and calcium ions. Hence, the modification of COP with readily polymerizable groups is necessary to broaden its comprehensive use. For example, Li et al. functionalized COP with adipic acid dihydrazide for wound healing.^[^ ^35^ ^]^ Besides, Yano et al. first developed glycidyl methacrylated collagen peptide (GMA‐COP) with photo‐crosslinking capacity, which showed good compatibility for fibroblasts in 2D culture models.^[^ ^36^ ^]^ However, the high concentration of photoinitiator (Irgacure 2959 at 0.5% (w/v)), high UV intensity (300 nm, 60 mW·cm^−2^) and long irradiation time (7.5 min) may produce potential cytotoxicity in 3D stem cells cultures and impede its 3D application.^[^ ^36^ ^]^ In this study, we established a new modified COPMA via reacting COP with methacrylate anhydride and investigated its 3D application. This COPMA showed a high degree of methacrylation (94.5%), resulting in rapid UV crosslinking capacity in mild curing conditions in terms of less photoinitiator (0.05–0.1% (w/v) of LAP), less intense UV light (20 mW·cm^−2^), less irradiation time (2–3 min). The storage modulus of COPMA hydrogels could be modulated from 0.35 ± 0.035 kPa to 156 ± 8.1 kPa by simply adjusting the concentration of COPMA from 15% to 50% (w/v). These COPMA hydrogels show improved mechanical properties compared to collagen hydrogel (typical G′ ∼100 Pa).^[^ ^37^, ^38^ ^]^

We initiated double networks between COPMA and XG to enable bioprinting of COPMA and stabilize the bioprinting constructs. Herein, XG not only acted as a thickener to endow the COPMA with shear thinning properties but also increased the mechanical properties of COPMA‐XG compared to COPMA alone at the same concentration. The COPMA_15_‐XG_3.5_ hydrogels showed a more stable structure with 22.8% degradation over 28 days, when compared to calcium ion cross‐linked COP‐alginate hydrogels from literature.^[^ ^39^ ^]^ The latter shows COP release up to 50% over 250 h via gradual breakage of the amide and ionic bonds.^[^ ^39^ ^]^ Besides, the XG hydrogel from literature studies mainly contains ionic crosslinks, which could lead to erratic XG leakage and hydrogel instability.^[^ ^22^ ^]^ Le et al. reported a bioink containing Fe (III) and XG, which exhibited a significant weight loss of 60–80% after 21 days of utilization at varying iron concentrations.^[^ ^40^ ^]^ However, our UV curable COPMA_15_‐XG_3.5_ bioink showed superior curing properties (remained gel fraction 77.2% ± 2.2% at day 28) compared to the former ionically crosslinked XG bioink. Besides, COPMA‐XG bioinks show flexible printability at room temperature and physical temperature, compared to the thermosensitive GelMA and collagen bioinks that need to be bioprinted at low temperature or in supporting baths.^[^ ^41^, ^42^ ^]^ The COPMA‐XG bioinks show similar stability to GelMA (∼23% degradation in 28 days),^[^ ^43^ ^]^ but better stability than collagen bioinks (∼40% degradation in 21 days).^[^ ^44^ ^]^ The COPMA‐XG bioinks display similar good biocompatibility and bioactivities to GelMA and collagen‐based bioinks,^[^ ^10^, ^45^ ^]^ such as promoting cell proliferation and differentiation, and producing ECM. Overall, bioprinted cell‐laden COPMA‐XG hydrogels show more accessible good printability and better mechanical properties than collagen‐based constructs,^[^ ^41^, ^42^ ^]^ and better biocompatibility and bioactivity than synthetic non‐biodegradable constructs.^[^ ^46^ ^]^

The hydrogen bonding of XG with COPMA was interspersed in a dense COPMA covalent network, resulting in synergistic interactions through physical and chemical crosslinking. This interpenetrating network endowed COPMA_15_‐XG_3.5_ hydrogels with self‐healing capabilities. Compared to other self‐healable natural hydrogels (such as chitosan‐based and hyaluronic acid‐based hydrogels),^[^ ^47^, ^48^ ^]^ the natural COPMA‐XG hydrogels show better stability (∼23% degradation in 28 days) than chitosan–fibrin hydrogels (degrading ∼70% in 14 days).^[^ ^49^ ^]^ Compared to self‐healable synthetic hydrogels, such as PEG‐based,^[^ ^50^ ^]^ and benzene‐1,3,5‐tricarboxamide‐based hydrogels,^[^ ^46^ ^]^ COPMA‐XG hydrogels show similar tunable stiffness and self‐healing properties, yet better biocompatibility with higher cell viability and cell spreading. The self‐healing properties of constructs may lay a foundation for keeping integration of constructs and tissues during regeneration.^[^ ^51^ ^]^ The mechanism of self‐healing of COPMA‐XG constructs mainly contributes to dynamic hydrogen bonding, which also widely exists in human tissues. Thus, these constructs may interact with the tissue in the dynamic microenvironment, protect tissue as buffer by resorting damage, and provide injured tissue with ECM‐mimic constructs with numerous cells to accelerate regeneration.

We bioprinted hMSCs containing COPMA‐XG hydrogels that provides a proof‐of‐concept study to explore the potential of COP‐based constructs in ligament regeneration. COP has been reported to exert a beneficial impact on the alleviation of pain associated with osteoarthritis and the enhancement of joint physical functionality.^[^ ^16^, ^52^ ^]^ However, the biomedical applications of COP remain in oral and injectable supplements.^[^ ^53^ ^]^ There are a few studies using COP in biofabrication strategies, and no report about COP‐based constructs for ligament tissue engineering.^[^ ^34^, ^35^ ^]^ In this study, we enabled bioprinting of COP‐based hydrogels and investigated its potential in ligament regeneration. The differentiation of hMSCs in the COPMA_15_‐XG_3.5_ construct was verified by increased deposition of GAG and total collagen, along with expression of COL‐I and scleraxis over time both in proliferation and differentiation media. COPMA_15_‐XG_3.5_ construct as a versatile matrix can support cell proliferation and differentiation, and the differentiation effect was amplified in the differentiation conditions supplemented with TGF‐β3. The growth factor can be changed to induce stem cells differentiation to different cell phenotypes to meet different application requirements.

These hMSCs laden COPMA‐XG constructs could act as cell depot delivery system for tissue engineering. One of the main issues with tissue regeneration is the lack of abundant cellular sources.^[^ ^54^ ^]^ Therefore, we first bioprinted numerous hMSCs in the COPMA_15_‐XG_3.5_ constructs for ligament regeneration. We noticed that the encapsulated hMSCs displayed an increased metabolic activity, high viability, and cell spreading in constructs over 28 days. This indicated the good biocompatibility of the constructs and good cell‐hydrogel interactions. Interestingly, the cell number in the bioprinted COPMA_15_‐XG_3.5_ constructs decreased while rising in the well plate, indicating cell delivery from hydrogels. Consistent with the report of Yang et al, COP could contribute to fibroblast proliferation and migration.^[^ ^55^ ^]^ The COPMA_15_‐XG_3.5_ constructs may be able to act as an effective cell carrier for cell‐based therapies to deliver the desired cells from the constructs to the defected tissue, which might improve tissue integration. Subsequently, we developed COPMA_20_‐XG_3.5_ hydrogels with higher stiffness to compare the cell spreading and migration behaviors with COPMA_15_‐XG_3.5_ hydrogels. hMSCs performed distinct behaviors in the COPMA_20_‐XG_3.5_ constructs, including maintenance of the original round shape with stable cell density and position. This may be advantageous for the extended application of COPMA_20_‐XG_3.5_ constructs in cartilage regeneration that requires a round cell phenotype rather than an elongated phenotype.

For bioprinted cell‐laden natural hydrogels for ligament regeneration, it is a challenge to obtain both comparable mechanical properties and 3D cell‐laden hydrated ligament‐like ECM‐rich constructs. Thus, there is still no reported bioprinted construct that possesses both the aforementioned superior properties. Our bioprinted COPMA‐XG constructs, as natural‐based ECM‐mimic constructs, can promote hMSCs to remodel ECM such as ligament‐like matrix with abundant ECM, COL‐I, and SCX. Although the bioprinted cell‐laden COPMA‐XG hydrogel is still not able to reach the mechanical requirement for ligament, as a natural bioink, it shows self‐healing properties, higher mechanical properties, better bioprintability and stability than collagen bioinks, and can act as cell depot to support cell proliferation and differentiation in vitro. It shows potential as a collagen bioink alternative to address its bioprinting problem and simplify the bioprinting process. Further research is required to further improve the mechanical properties of these constructs, monitor their mechanical properties during ECM remodeling and assess the ligament regeneration in vivo.

## 4 Conclusion

Although COP shows promising biomedical effects, research on COP and its clinical applications are limited to oral and injectable supplements. Its low viscosity and inability to rapidly form hydrogels limit its application forms and broader utility in tissue engineering. In this study, we developed a novel readily UV‐curable COP‐based hydrogels and bioprinted hMSCs laden COP‐based hydrogels with interpenetrating networks. Thanks to the synergetic effect of dual physical and chemical crosslinking in COPMA‐XG hydrogel networks, the hydrogel showed tunable mechanical properties, good printability, and maintained stability in the medium up to 100 days. Moreover, the hydrogel showed self‐healing properties due to the formation of dynamic bonding (e.g., hydrogen bonding). Additionally, we investigated the ability of bioprinted COPMA‐XG constructs to support hMSCs proliferation and differentiation toward the ligament phenotype. hMSCs spread throughout the 3D bioprinted constructs with high viability and produced ECM, COL‐I, and SCX in both proliferation and differentiation media over 28 days, indicating good cell‐hydrogel interactions in biomimetic constructs. Synergic effect between biomimetic constructs and TGF‐β3 further promoted hMSCs differentiation toward ligaments in constructs under differentiation conditions. Overall, this study provided a solution to bioprint COP‐based hydrogels and showed the versatility of bioprinted COPMA‐XG constructs with potential for ligament regeneration. This straightforward strategy of bioprinting COP‐based constructs could pave the way to develop advanced hydrogels with multiple functionalities and broaden their applications in tissue regeneration.

## 5 Experimental Section

### Development of COPMA

COPMA was developed by the optimized protocol, adapted from Tytgat et al.^[^ ^56^ ^]^ Briefly, fifty grams of COP (Viscofan, Germany) was dissolved in 500 mL of phosphate buffer (0.1 m, pH 7.8). Consequently, methacrylic anhydride (1.3 equiv with respect to the primary amines present in COP, 42.6 mmol, 6.71 mL) was added dropwise. The mixture was reacted at 40 °C for 3 h with continuous stirring, followed by centrifugation to remove the insoluble part. Afterward, COPMA was dialyzed (MWCO 1 kDa) against distilled water at 37 °C for 24 h to eliminate excess of unreacted methacrylic anhydride and the methacrylic acid produced during the reaction. Finally, the COPMA was obtained by lyophilized for 1 week to get yellowish fluffy solid with a yield of 40%. The modification was confirmed by ^1^H NMR spectroscopy (Bruker Avance III HD 300 MHz, Germany) with D_2_O as solvent, and Fourier‐transform infrared spectroscopy (FTIR, Nicolet iS50FT‐IR, Thermo Scientific, USA) with 32 scans between 400 and 4000 cm^−1^ and 0.5 cm^−1^ resolution. The molecular weight of COPMA was measured by aqueous gel permeation chromatography (GPC) using a Prominence LC‐2030C system (Shimadzu, Japan) equipped with a refractive index detector and a photodiode array detector, connected to Shodex SB‐803 HQ & SB‐804 HQ columns (Showa Denko, Japan). The analysis was performed with 0.1 m sodium nitrate as the eluent at a flow rate of 0.4 mL min^−1^, and poly(ethylene glycol) standards were used to calibrate the molecular weight.

### Quantification of Free Primary Amines and Degree of Substitution

To determine the free primary amines in COP and COPMA and the degree of substitution (DS) of COPMA, an ortho‐phthalic dialdehyde (OPA) assay was applied according to Tytgat et al.^[^ ^56^ ^]^ Briefly, stock solution 1 was prepared by dissolving 10 mg of OPA (Sigma–Aldrich) in 5 mL of ethanol and then diluting it to 20 mL with MilliQ water. Stock solution 2 was prepared by dissolving 12.5 µL of 2‐mercaptoethanol (Sigma–Aldrich) in 25 mL of a borate buffer (0.1 m, pH 10). A series of n‐butylamine (Sigma–Aldrich) standard solutions (0, 0.002, 0.004, 0.006, 0.008, and 0.01 m) were prepared. COP and COPMA (20 mg mL^−1^) were dissolved in MilliQ water. To a 96‐well plate, MilliQ water (63.4 µL), stock solution 2 (100 µL), sample or standard solution (3.33 µL), and stock solution 1 (33.4 µL) were added subsequently. Immediately, the absorbance at 335 nm was measured by a CLARIO star plate reader (BMG Labtech, Germany). The DS of COPMA was calculated by formula 1:

(1) $$DS\left(\%\right)=\frac{\mathrm{primary}\;Amines\left(COP\right)-\mathrm{primary}\;Amines\left(COPMA\right)}{\mathrm{primary}\;Amines\left(COP\right)}\times 100$$

### Transmission Electron Microscopy (TEM)

COPMA was dissolved in DPBS at 0.5% (w/v) then filtered through a 0.22 µm PES syringe filter. After keeping this solution at 25 °C for 72 h, a 200‐mesh carbon‐coated copper grid was placed on a 10 µL droplet of COPMA. After washing with distilled water, the grid was then placed on filter paper to dry overnight. Uranyl acetate was used to stain the TEM grids using negative‐staining techniques. Finally, samples were imaged by TEM (FEI electron microscope, USA).

### Development of COPMA‐XG Formulations

COPMA was dissolved in modified Dulbecco's phosphate‐buffered saline (DPBS, without calcium chloride and magnesium chloride, Sigma‐Aldrich) at a concentration of 30% (w/v) at room temperature and sterilized by filtering through a 0.22 µm polyethersulfone (PES) syringe filter (Sigma‐Aldrich). Lithium phenyl‐2,4,6‐trimethyl‐benzoyl phosphinate (LAP, Sigma‐Aldrich) photoinitiator stock solution (4% (w/v)) was dissolved in DPBS, then filtered by 0.22 µm PES syringe filter. XG (Sigma‐Aldrich) powder was sterilized by fast autoclaving at 134 °C, 4 min cycle in pre‐vacuum sterilizer (AH‐21‐S2‐DRY, Raypa, Spain). After cooling to room temperature, it was dissolved in sterile DPBS at concentrations of 3%, 5% and 7% (w/v) and kept rolling overnight on a roller tube platform. Finally, these XG stock solutions were stored at 4 °C until use.

Six formulations of COPMA‐XG hydrogels were developed, varying in mass ratio of COPMA and XG (Figure S5, Supporting Information). The sterilized COPMA solution (with final concentration of 0.1% LAP) was mixed with XG by gel‐loading pipette. For example, COPMA_15_‐XG_3.5_ was mixed by 0.5 mL of 30% COPMA stock solution with 0.5 mL of 7% XG stock solution, to get final concentration of 15% COPMA‐3.5% XG (mass ratio of 4.3:1). A vial inversion test was conducted, and photos were taken at 5 min and 24 h to observe viscosity and flow behavior.

### 3D Printing of COPMA‐XG Hydrogels

According to the inverse vial test, three hydrogels that did not flow were further 3D printed into a square model (1 cm × 1 cm, wide×length) with a meandering pattern by 3D extrusion bioprinter (BioScaffolder 3.1, Gesim, Germany). The printing parameters were developed (shown in Table S1, Supporting Information), varying speed from 30 to 50 mm s^−1^ and pressure from 30 to 45 kPa. The extruded constructs were printed through a 25 G nozzle tip (0.25 mm diameter, SmoothFlow Tapered Dispense Tips, Nordson, USA), followed by exposing under UV light (UVP CL‐1000 Ultraviolet Crosslinkers, Analytik Jena, Germany) at 365 nm, 20 mW·cm^−2^ for 3 min.

### Scanning Electron Microscopy (SEM)

The morphology of the hydrogels was investigated by SEM. Hydrogels were initially frozen at −30 °C, followed by freeze‐drying. Subsequently, they were mounted on an aluminum sample holder using a conductive double‐sided adhesive. After gold coating by C150TES sputter coater (Quorum, United Kingdom), samples were imaged with a SEM (Jeol JSM‐IT200 InTouchScopeto, Japan) at an accelerating voltage of 10 kV.

For the cryo‐scanning electron microscope (cryo‐SEM), the hydrogel was cooled in liquid ethane and transferred to liquid nitrogen. The frozen hydrogel was transferred to the cryo‐SEM (Aquilos I, Cryo‐FEI, Thermo Fisher) and sputter‐coated using the in‐built platinum coater, and then imaged at an accelerating voltage of 2 kV.

### Rheological and Viscosity Tests

Rheological tests were conducted at 20 °C using a DHR‐2 rheometer (TA Instruments, USA) with a cone‐plate setup (20 mm diameter at a 2.002° with a gap of 53 µm). First, oscillatory time sweeps at 2% strain and 10 rad·s^−1^ were conducted for 5 min. The storage modulus (G′) and loss modulus (G″) were continuously monitored, allowing for the observation of rheological dynamics during photopolymerization. Specifically, after equilibration for 1 min, the hydrogel was exposed to UV light (M365LP1 LED, with DC2200 LED Driver modulation, Thorlabs, Germany) at 365 nm, 20 mW·cm^−2^ for 4 min. Consequently, a frequency sweep was performed over 1.0 to 100 rad·s^−1^ with a constant strain of 2% in the oscillation mode. Finally, oscillatory strain amplitude sweep was carried out from 0.1% to 10% strain at 10 rad·s^−1^ frequency. To evaluate the viscosity of COPMA‐XG, shear rate sweeps were conducted across 0.1 to 1000 s^−1^ at room temperature. All rheological and viscosity tests were conducted at least twice.

### Self‐Healing Tests

For the self‐healing tests, strain sweep curves were recorded from 0.1% to 500% strain. The cyclic strain sweep curves were recorded under 1% strain and 500% strain alternatively. The hydrogels were formed in a mold (8mm*1 mm, diameter*depth) with mixing hydrogel precursor with or without rhodamine B (red) and trypan blue (blue) to get hydrogels in red, blue, or original colorless color. After UV crosslinking, all hydrogels were cut into 2 pieces and recombined 2 pieces of different color hydrogels at room temperature for 3 h to assess self‐healing.

### Compressive Tests

Hydrogel cylinders (10 mm diameter) were compressed at a strain rate of 0.04 mm·s^−1^ until break or a maximum compression of 80% was reached by TA ElectroForce system (TA Instruments, USA) equipped with a 450 N load cell. Prior to testing, the height and diameter of each sample were measured with a caliper.

### Swelling Ratio Determination

After getting the initial weight (W_0_), hydrogels were swollen at 37 °C for 1 and 28 days in PBS. Consequently, hydrogels were gently dipped with tissue paper to obtain the swollen weight (W_s_). The swelling ratio was defined as:

(2) $$Swellingratio\;\;\left(\%\right)=\frac{Ws-W0}{W0}\times 100$$

### Gel Fraction Determination

Hydrogels were freeze dried to get the initial dry weight (W_d0_). After swelling at 37 °C for 24 h in distilled water. These swollen hydrogels were lyophilized again to get weight (W_d1_). The gel fraction was calculated based on the equation:

(3) $$Gelfraction\;\;\left(\%\right)=\frac{Wd1}{Wd0}\times 100$$

### Analysis of Scaffold Shape Stability in Culture Medium

The stability of scaffolds in basic culture medium was assessed. Optical microscopy images were captured at 1, 28 and 100 days by fluorescence microscope (Eclipse Ti‐E, Nikon, Japan) to monitor degradation of the same samples over time.

### hMSCs Culture Media

Herein, two types of culture media were used: proliferation medium and ligament differentiation medium. The proliferation medium consisted of α‐MEM (Alpha minimum essential medium with GlutaMAX (Gibco), supplemented with 10% (v/v) fetal bovine serum (FBS, Sigma–Aldrich) and 1% (v/v) penicillin/streptomycin solution (Pen‐Strep, Thermo Fisher). The ligament differentiation medium was prepared fresh weekly, with the following formulation: 4.5 g L^−1^ high‐glucose DMEM (Gibco), 10% (v/v) fetal bovine serum (FBS, Sigma–Aldrich), 1% (v/v) penicillin/streptomycin solution (Pen‐Strep, Thermo Fisher), and 10 ng mL^−1^ transforming growth factor‐beta 3 (TGF‐β3, PeproTech).

### hMSCs Culture

Human bone marrow mesenchymal stem cells (hMSCs) from PromoCell (donor Caucasian woman, 30 years old) were cultured in T225 flasks at a density of 1000 cells cm^−2^ in proliferation medium. The cells were passaged when reaching 80% confluence and experiments were conducted using cells at passage 4.

### Bioprinting of hMSCs Laden Constructs

The hydrogel precursor was prepared by mixing 30% COPMA (with 0.1% LAP) and 7% XG in 1:1 (v/v) by gel pipette, followed by centrifuging at 800 rpm for 10 min to remove bubbles. Consequently, hMSCs pellet was obtained by trypsinization and centrifugation at 400 rpm for 4 min. The hydrogel precursor was immediately added into the hMSCs cluster, and then gently but thoroughly mixed with a 10 µL pipette tip to avoid bubbles. This bioink with 4 million/mL (4 m mL^−1^) of hMSCs was loaded into a 3 mL sterilized syringe with the assistance of centrifugation at 400 rpm for 1 min, then coupled with a precision tip with an internal diameter of 250 µm (Nordson, USA). The hydrogel was bioprinted into 3 layers (10 mm × 10 mm × 0.6 mm, wide × length × height) with 40 mm s^−1^ of extrusion speed and 45 kPa of pressure by BioScaffolder 3.1 (Gesim, Germany). The bioprinted constructs in a non‐treated polystyrene (PS) 12‐well plate were UV crosslinked by exposing under UV light (365 nm, 20 mW·cm^−2^) for 3 min. Consequently, cells laden constructs were cultured with 2 mL of required culture media at 37 °C under 5% of CO_2_ atmosphere. The above media were refreshed every 2 days.

### Quantification of Metabolic Activity

Metabolic activity was quantified by the Cell Titer‐Glo 3D assay (Promega). Equal volumes of reagent and culture medium were added to bioprinted constructs on day 0, 1, 7, 14, and 28, ensuring thorough mixing with the hydrogel debris by pipetting. Following a 30‐min incubation at room temperature, 100 µL of supernatant was transferred to a white bottom 96 well plate and luminescence was measured using a CLARIO star plate reader (BMG Labtech, Germany).

### Cell Viability Analysis

To assess the viability of cells within the bioprinted constructs, a live/dead assay was conducted. After removing the medium and rinsing by DPBS, constructs were immersed in 2 µm of calcein acetoxymethyl solution (Calcein AM, Thermo Fisher) for 25 min at 37 °C in the dark. Subsequently, ethidium homodimer‐1 solution (EthD‐1, Thermo Fisher) at final concentration of 0.06 µm was added to the wells and incubated for an additional 25 min at 37 °C. Finally, constructs were rinsed twice with DPBS and then imaged by a fluorescence microscope (Eclipse Ti‐E, Nikon, Japan).

### DNA Quantification

Samples were harvested after day 0, 1, 14, and 28 days of culture and stored at −80 °C until analysis. DNA extraction involved freeze‐thawing constructs three times in liquid nitrogen, followed by digested in 1 mg mL^−1^ proteinase K solution in 50 mm Tris/1 mm EDTA buffer at 56 °C for 16 h. After an additional three freeze‐thaw cycles in liquid nitrogen, the DNA content was quantified using the CyQuant cell proliferation assay kit (Thermo Fisher, USA) following the manufacturer's protocol. In brief, after incubating the samples for 2 h in lysis buffer containing RNase at room temperature, 100 µL of each sample (in triplicate) was mixed with 100 µL of 2× GR‐dye solution in a black 96‐well plate. After a 15‐min incubation, fluorescence was measured at 520 nm using a CLARIO star plate reader (BMG Labtech, Germany).

### Glycosaminoglycan (GAG) Quantification

GAGs were quantified from samples following the Proteinase K digestion step using a 1,9‐dimethylmethylene blue (DMMB, Sigma‐Aldrich) solution. Chondroitin sulfate from shark cartilage (Sigma–Aldrich) was used to establish a standard curve. Briefly, 150 µL of DMMB solution was mixed with 50 µL of sample and 5 µL of NaCl in a 96‐well plate. The absorbance was measured at 525 and 595 nm using a CLARIO star plate reader, and the difference between these readings was used for quantification. To keep in the linearity range, all hydrogel samples were diluted 100 times.

### Total Collagen (COL) Quantification

Total collagens were quantified from samples following the Proteinase K digestion step using a collagen assay kit (Sigma–Aldrich). In brief, 20 µL of sample or collagen standard was digested in 30 µL of master reaction buffer with collagen enzyme. As sample or standard blank, 20 µL of sample or standard was added to 30 µL of buffer without collagen enzyme. The above mixture in a black 96 well plate was incubated at 37 °C for 1 h, followed by addition incubation with 40 µL of dye reagent for 10 min. After another incubation with 8 µL of developer for 10 min at 37 °C, the florescence at 375 and 465 nm was measured in a CLARIO star plate reader, and the difference in florescence was used to quantify the collagen deposition. To keep in the linearity range, all hydrogel samples were diluted 1000 folds.

### Immunofluorescence

Constructs were fixed in 4% paraformaldehyde (PFA) after 1 and 28 days, followed by rinsing with PBS. Blocking and permeabilization were performed together overnight at 4 °C with 1% (w/v) Triton X‐100, 0.05% (w/v) Tween‐20, 5% (w/v) goat serum (Sigma–Aldrich), and 1% (w/v) Bovine Serum Albumin (BSA, VWR) in PBS. After remove the above solution, samples were incubated for 24 h at 4 °C with mouse anti‐collagen I (COL‐I, 1:200, ab6308, Abcam) and rabbit anti‐Scleraxis (SCX, 1:200, ab58655, Abcam) primary antibodies, followed by washed with washing buffer consisting of 1% BSA and 0.05% Tween‐20 in PBS. After incubation of secondary antibodies (goat anti‐mouse and goat anti‐rabbit, both at 1:500 in washing buffer, Abcam) conducted at 4 °C overnight, samples were washed 3 times with PBS. Finally, F‐actin was stained by Alexa Fluor Phalloidin 568 (1:75, Thermo Fisher) in PBS for 1 h and DNA was stained by DAPI for 20 min in the dark at room temperature. After rinsing with PBS, samples were imaged with a confocal microscope (Leica TCS SP8 CARS, Germany). The relative fluorescence area (%) was calculated by Image J.

### Statistical Analysis

Statistical analysis was performed using GraphPad Prism software (version 10.3, USA). Student's t‐test, one‐way and two‐way ANOVA with Tukey's multiple comparison test were used to assess statistical significance. All assays were conducted at least in triplicate, unless otherwise specified in the above sections. Data are expressed as mean ± standard deviation (SD) or shown with SD bars in the graphs.

## Conflict of Interest

The authors declare no conflict of interest.

## Supporting information



Supporting Information

## Data Availability

The data that support the findings of this study are available from the corresponding author upon reasonable request.
