# Supplementary material for: Mechanical Reinforced and Self‐healing Hydrogels: Bioprinted Biomimetic Methacrylated Collagen Peptide‐Xanthan Gum Constructs for Ligament Regeneration
Source: Adv Healthc Mater. 2025 Jul 16;14(25):2502341. doi: 10.1002/adhm.202502341 (PMC12477574; doi:10.1002/adhm.202502341)
Supplement: Supplementary file 1 — Supporting Information [file ADHM-14-0-s001.pdf]

ADVANCED HEALTHCARE MATERIALS

Supporting Information

for *Adv. Healthcare Mater.*, DOI 10.1002/adhm.202502341

Mechanical Reinforced and Self-healing Hydrogels: Bioprinted Biomimetic Methacrylated Collagen Peptide-Xanthan Gum Constructs for Ligament Regeneration

*Hongjuan Weng, Monize Caiado Decarli, Lei He, Wen Chen, Sabine van Rijt, Katrien V. Bernaerts and Lorenzo Moroni**

Supporting information

Mechanical Reinforced and Self-healing Hydrogels: Bioprinted Biomimetic Methacrylated Collagen Peptide-Xanthan Gum Constructs for Ligament Regeneration

Hongjuan Weng[1,2], Monize Caiado Decarli[1,3], Lei He[4], Wen Chen[1], Sabine van Rijt[4], Katrien V. Bernaerts[2], Lorenzo Moroni[1*]

1 Complex Tissue Regeneration Department, MERLN Institute for Technology Inspired Regenerative Medicine, Maastricht University, Maastricht, 6229 ER, The Netherlands
2 Sustainable Polymer Synthesis group, Aachen-Maastricht Institute for Biobased Materials, Maastricht University, Geleen, 6167 RD, The Netherlands
3 Department of Biomaterials and Biomedical Technology, University Medical Center Groningen, University of Groningen, Groningen, 9713 AV, The Netherlands
4 Instructive Biomaterials Engineering Department, MERLN Institute for Technology-Inspired Regenerative Medicine, Maastricht University, Maastricht, 6229 ER, The Netherlands

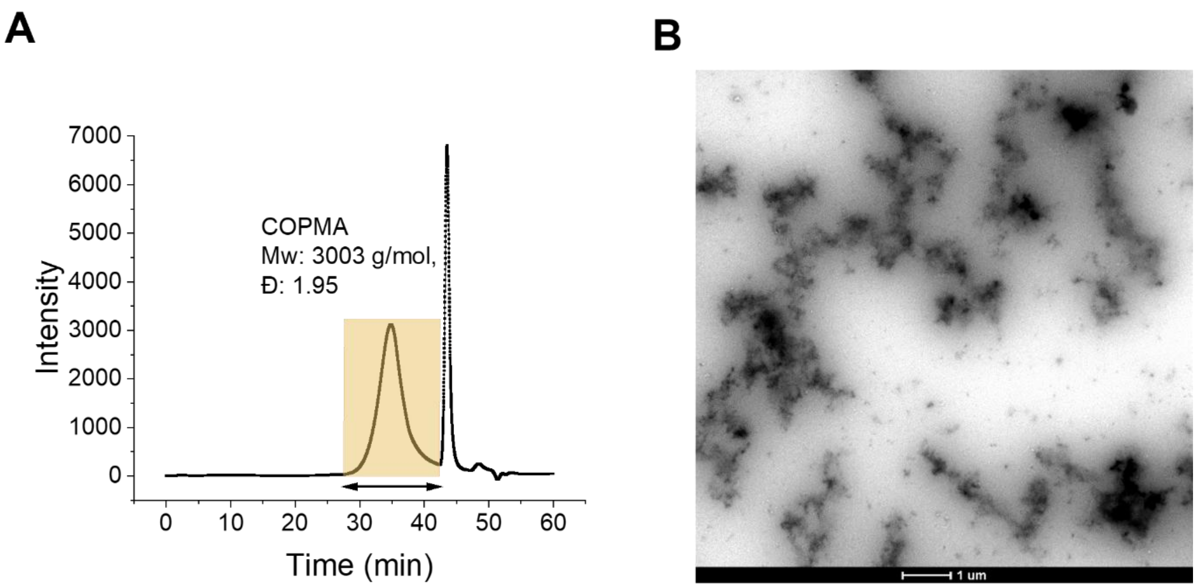

Figure S1. A) Molecular weight and dispersity of COPMA were measured by GPC. B) Negatively stained images of the COPMA in phosphate buffer at a concentration of 0.5% (w/v) and stained with uranyl acetate. Scale bar: 1 µm.

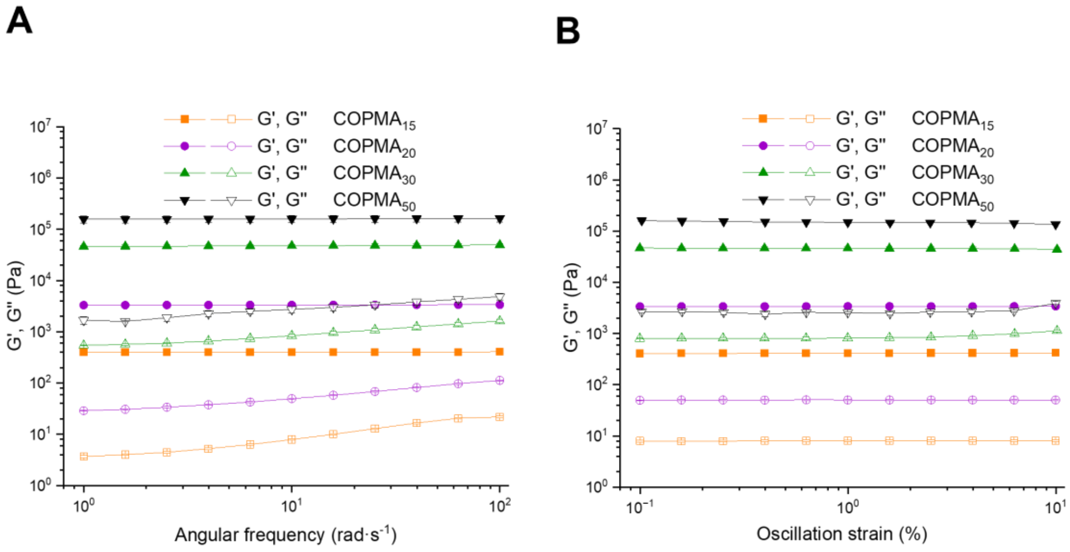

Figure S2. A) Rheological measurements of COPMA hydrogels obtained from frequency sweeps from 1 to 100 rad·s^{-1}, with a constant strain of 2%. B) Rheological measurements of COPMA hydrogels in strain sweep mode from 1% to 10% with a constant frequency of 10 rad·s^{-1}.

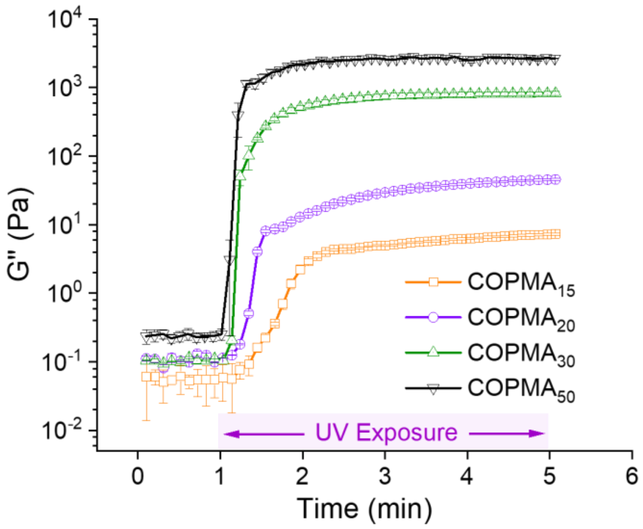

Figure S3. Loss modulus (G″) of COPMA hydrogels in various concentrations (15%, 20%, 30% and 50% (w/v)).

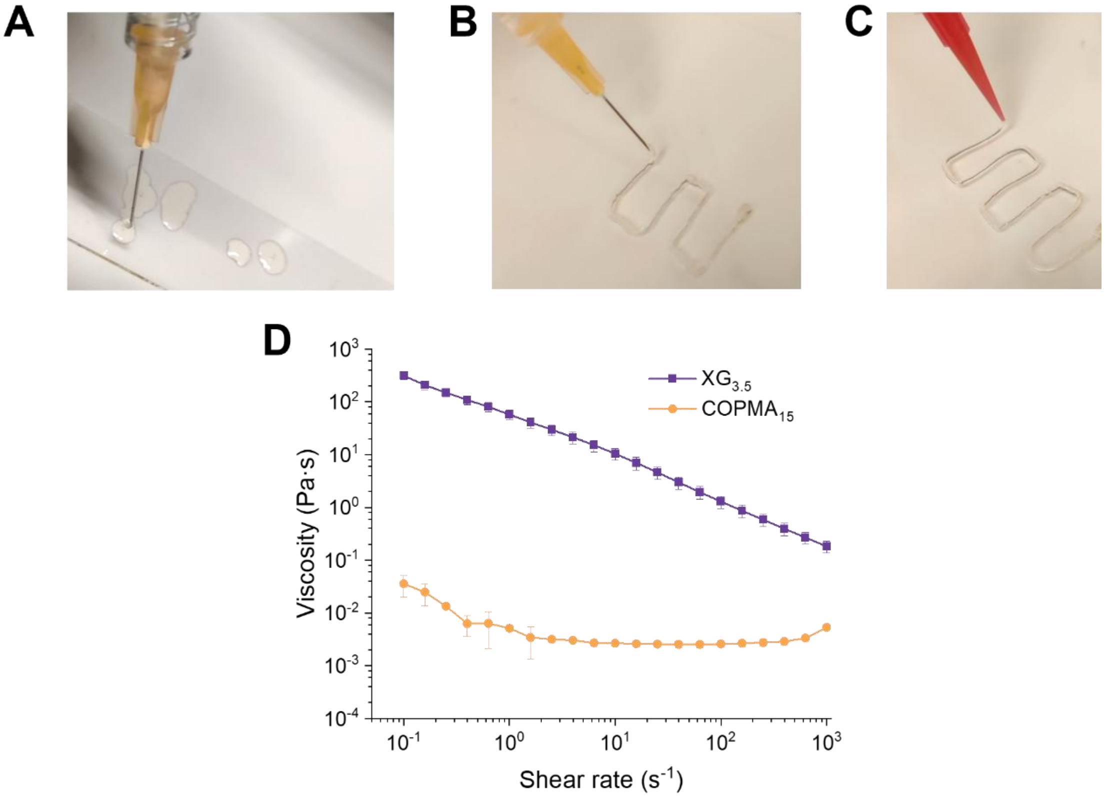

Figure S4. A) Poor printability of COPMA through 30 G nozzle (0.159 mm diameter). B-C) Good printability of COPMA-XG through a 30 G nozzle (0.159 mm diameter) and 25 G nozzle tip (0.25 mm diameter). D) Viscosity of 3.5% XG and 15% COPMA from 0.1-1000 s^{-1} shear rate.

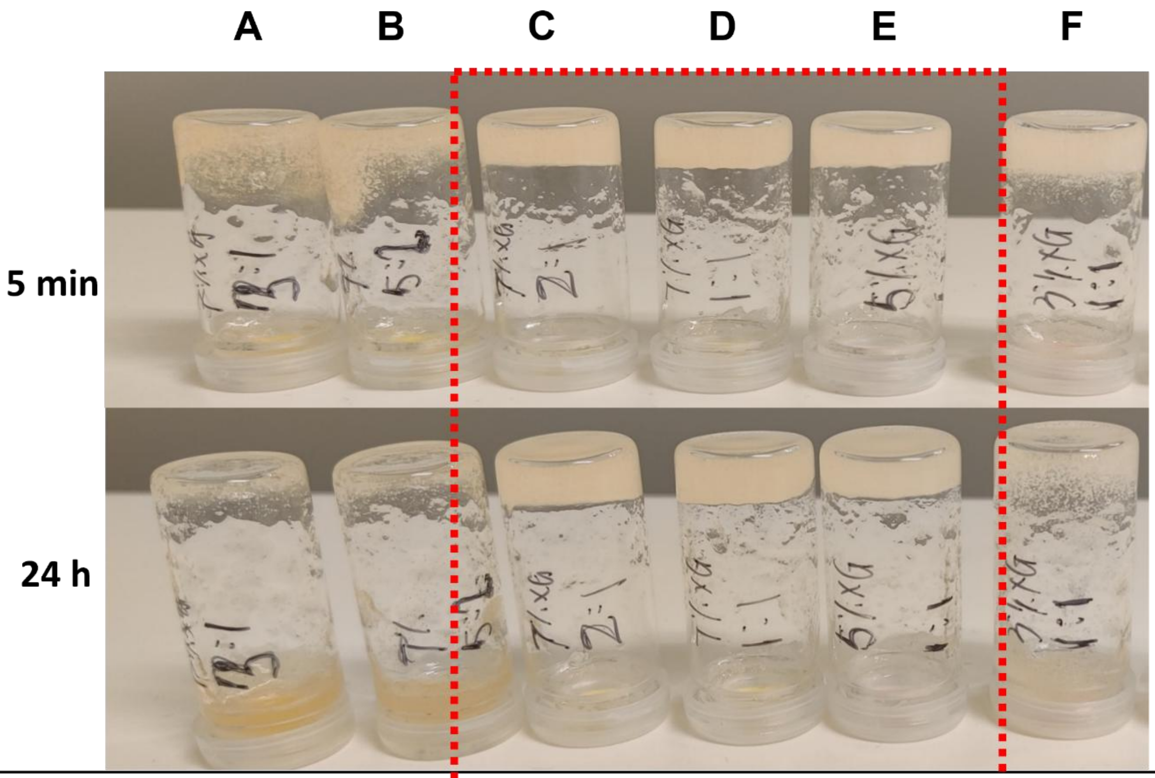

Sample code	$COPMA_{23}$-$XG_{1.8}$	$COPMA_{21}$-XG_{2}	$COPMA_{20}$-$XG_{2.3}$	$COPMA_{15}$-$XG_{3.5}$	$COPMA_{15}$-$XG_{2.5}$	$COPMA_{15}$-$XG_{1.5}$
Stock solutions (w/v)	30% COPMA+7% XG		30% COPMA+7% XG		30% COPMA+5% XG	30% COPMA+3% XG
Volume ratio (v/v)	3:1	5:2	2:1	1:1	1:1	1:1

Figure S5. Flow test of various mass ratios of COPMA and XG after 5 min and 24 h. The corresponding formulations (below). The red frame indicates the preferred formulations.

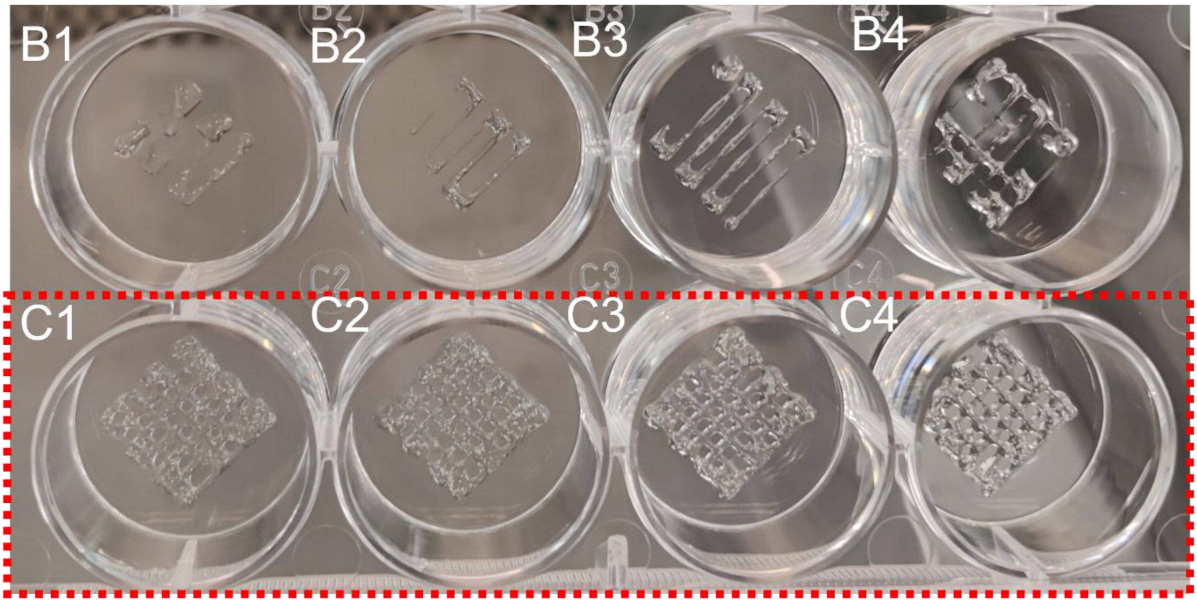

Well plate position	Speed [mm/s]	Pressure [kPa]	Radius [mm]	Layers	Strain height [mm]	Height [mm]
B1	30	30	6	1	0.2	0.2
B2	40	40	6	1	0.2	0.2
B3	40	40	8	1	0.2	0.2
B4	40	40	8	2	0.2	0.4
C1	35	40	8	2	0.2	0.4
C2	35	45	8	2	0.2	0.4
C3-C4	40	45	8	2	0.2	0.4

Figure S6. Optimization of 3D printing parameters. Images of 3D printed $COPXG_{15}$-$XG_{3.5}$ hydrogel (top). The corresponding printing parameters (below). The red frame indicates the optimized parameters.

Table S1. Optimized 3D printing parameters of COPMA-XG hydrogels ($COPMA_{15}$-$XG_{3.5}$, $COPMA_{15}$-$XG_{2.5}$ and $COPMA_{20}$-$XG_{2.3}$).

Formulations	Speed [mm/s]	Pressure [kPa]	Radius [mm]	Layers	Strain height [mm]	Height [mm]
$COPXG_{15}$-$XG_{3.5}$	35-40	40-45	8	5	0.2	1.0
$COPXG_{15}$-$XG_{2.5}$	30-40	30-35	8	5	0.2	1.0
$COPXG_{20}$-$XG_{2.3}$	40-50	30-35	8	5	0.2	1.0

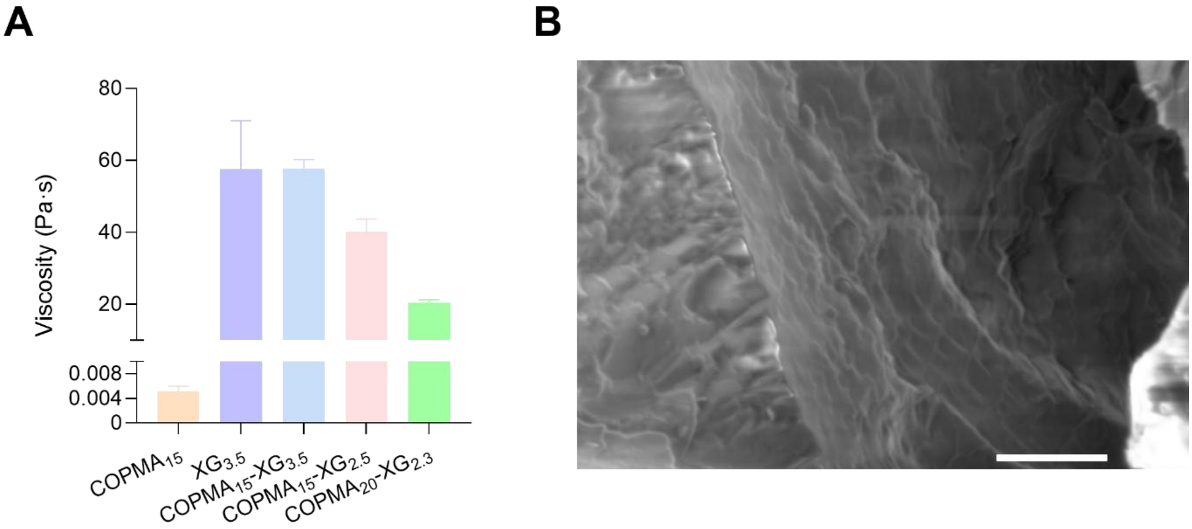

Figure S7. A) Viscosity of $COPMA_{15}$, $XG_{3.5}$ and COPMA-XG hydrogel precursor at a shear rate of 1 s^{-1}. B) Cryo-SEM image of COPMA-XG hydrogel, scale bar: 5 μm.

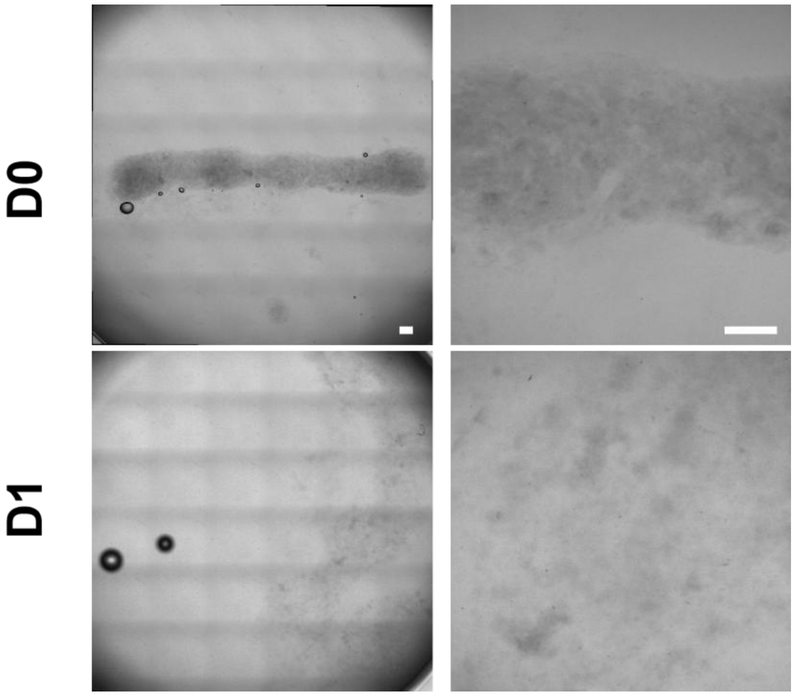

Figure S8. Optical images of $XG_{3.5}$ in culture medium on day 0 and day 1, scale bar: 500 μm.

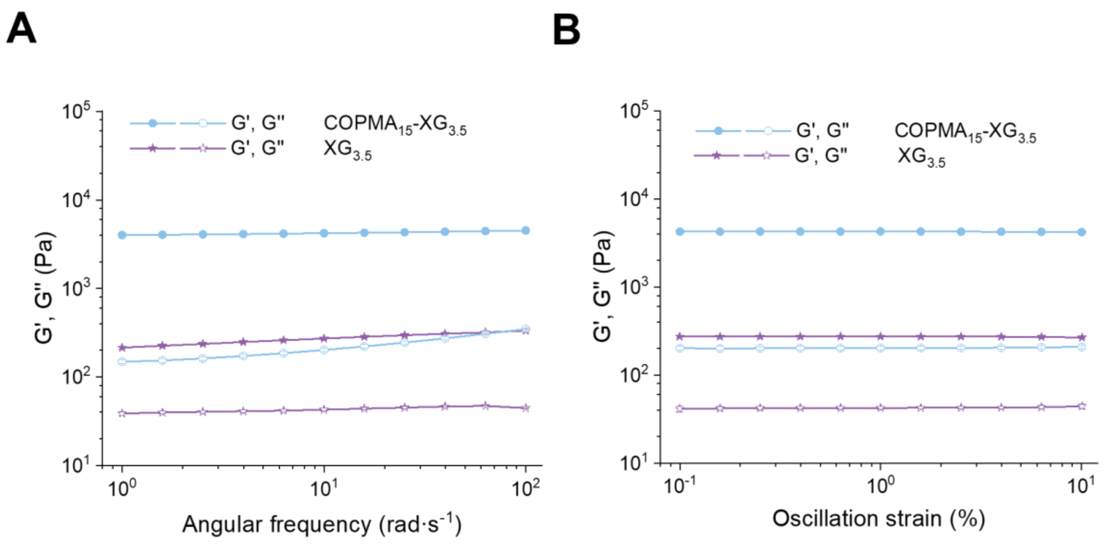

Figure S9. Rheological measurements of $COPMA_{15}$-$XG_{3.5}$ hydrogels and $XG_{3.5}$ in frequency sweep mode from 1 to 100 rad·s^{-1}, with a constant strain of 2%. B) In strain sweep mode from 1 to 10% with a constant frequency of 10 rad·s^{-1}.

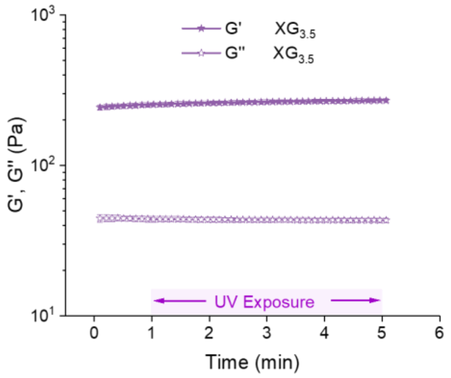

Figure S10. Photorheology of $XG_{3.5}$ in time sweep mode with 2% strain and 10 rad·s^{-1}.

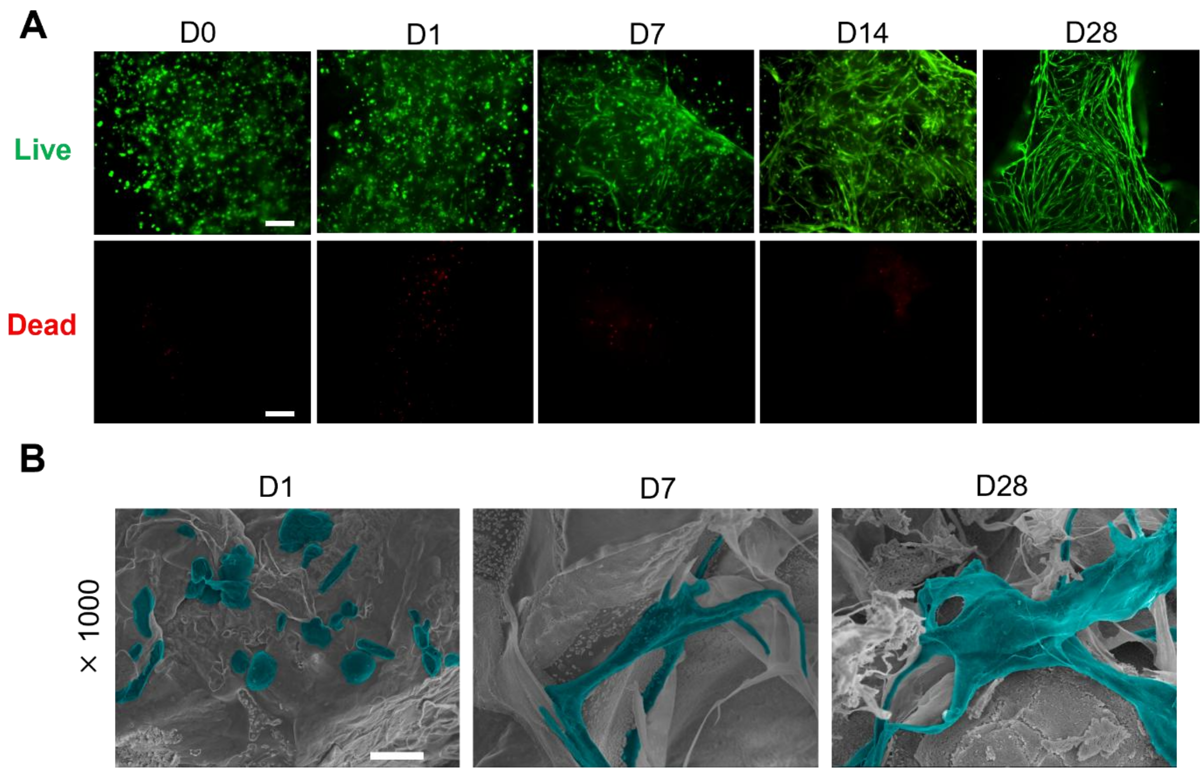

Figure S11. A) Live/dead staining of constructs in the proliferation medium, scale bar: 200 µm. B) SEM of hMSCs encapsulated in COPMA$_{15}$-XG$_{3.5}$ hydrogels on day 1, day 7, and day 28 at ×1000 magnification. The blue highlight indicates hMSCs. Scale bar: 20 µm.

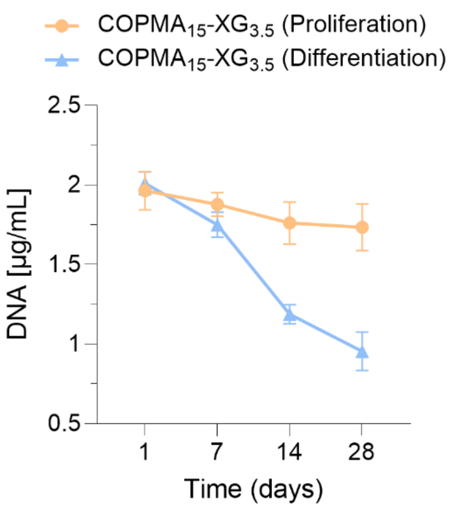

Figure S12. DNA in hMSC encapsulated $COPMA_{15}$-$XG_{3.5}$ hydrogels on day 1, 7, 14 and 28.

Table S2. Formulations of $COPMA_{20}$-$XG_{3.5}$ hydrogels.

Sample code	$COPMA_{20}$-$XG_{3.5}$
Stock solutions (w/v)	40% COPMA+7% XG
Volume ratio (v/v)	1:1

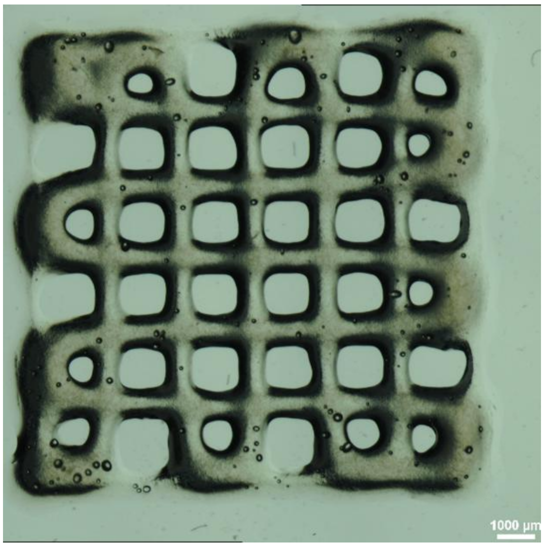

Figure S13. Optical image of bioprinted hMSCs laden $COPMA_{20}$-$XG_{3.5}$ hydrogel. Scale bar: 1000 μm.

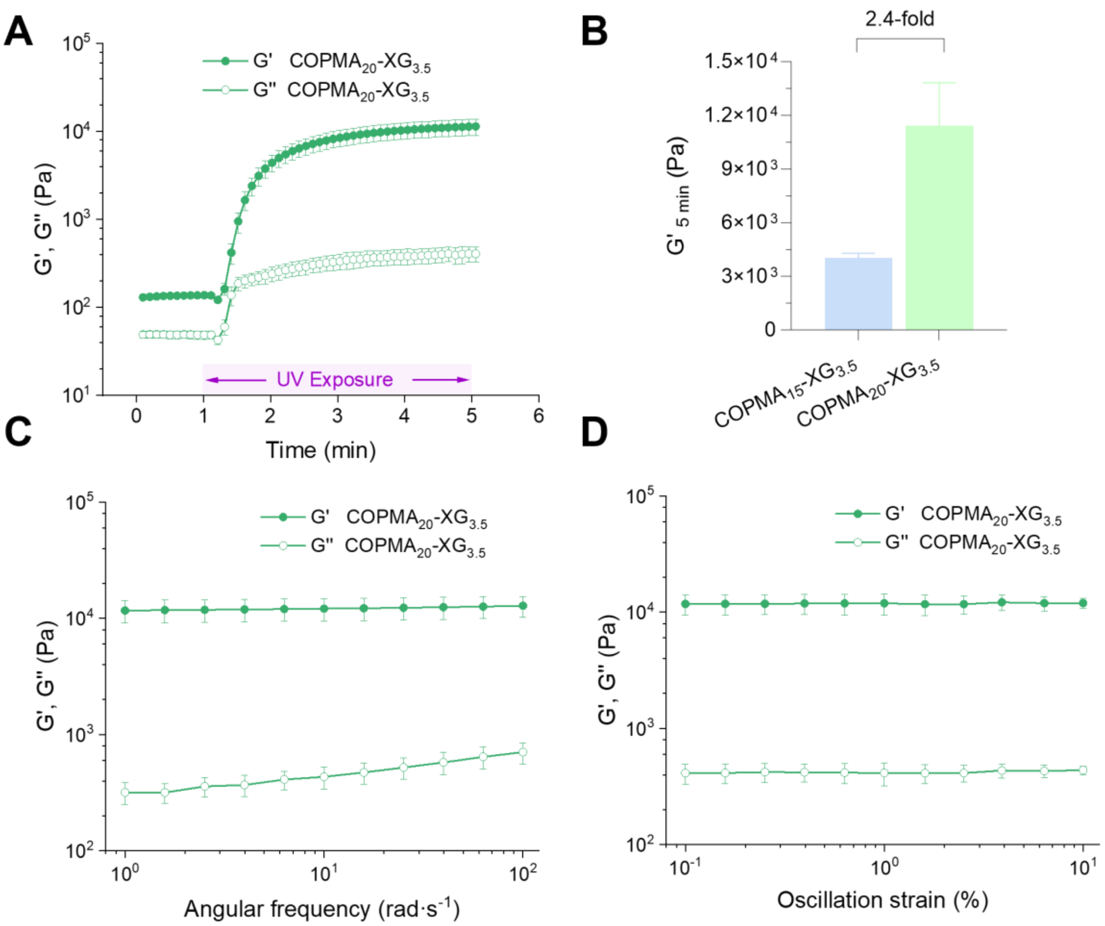

Figure S14. A) Photorheology of COPMA20-XG3.5 hydrogels. B) Comparison of storage modulus of COPMA15-XG3.5 and COPMA20-XG3.5 hydrogels after UV exposure. C) Rheological measurements of COPMA20-XG3.5 hydrogels in frequency sweep mode from 1 to 100 rad·s^{-1}, with a constant strain of 2%. D) Rheological measurements of COPMA20-XG3.5 hydrogels in strain sweep mode from 1 to 10% with a constant frequency of 10 rad·s^{-1}.

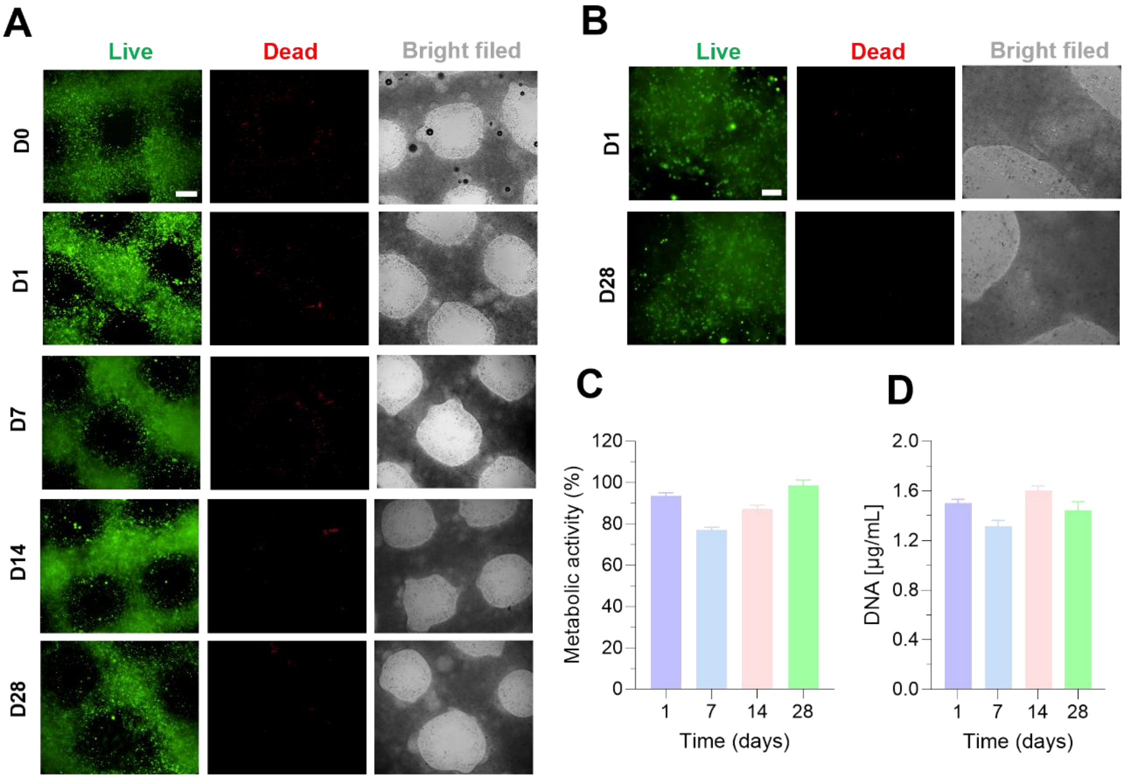

Figure S15. Biocompatibility of $COPMA_{20}$-$XG_{3.5}$ hydrogels in proliferation medium. A) Live/dead staining on day 0, 1, 7, 14, and 28 at ×4 magnification. Scale bar: 500 μm. B) Live/dead staining on day 1 and 28 at ×10 magnification. Scale bar: 200 μm. C) Metabolic activity. D) DNA quantification.
